# Periodic performance feedback and cognitive ability interactively mitigate the vigilance decrement

**DOI:** 10.3758/s13414-026-03314-8

**Published:** 2026-08-20

**Authors:** Matthew K. Robison, Reyes Flores, Benjamin Rudd

**Affiliations:** 1 Department of Psychology, University of Notre Dame, Notre Dame, IN USA; 2 Department of Psychology, The University of Texas at Arlington, Arlington, TX USA; 3 Department of Psychology, The University of Texas at Austin, Austin, TX USA

**Keywords:** Vigilance; Sustained attention; Feedback

## Abstract

We investigated the effects of periodic performance feedback across three experiments. In Experiment 1, participants were assigned to either a feedback condition, in which there was periodic performance feedback (*n* = 302), or to a control condition, which received no feedback (*n =* 293). Whereas the control condition demonstrated a large vigilance decrement, even over a brief task duration, participants in the feedback condition performed both better overall and demonstrated a significantly shallower decrement. Experiment 2 (*n* = 103) was a preregistered replication with an extended task duration, and we directly replicated the findings. Experiment 3 was another preregistered experiment (*n* = 283), in which we examined three conditions with the extended duration: feedback for the entire task, feedback for only the first half of the task, and no feedback for the entire duration. The conditions did not differ in performance. However, in a post hoc cross-experimental analysis, we discovered significant moderation of the feedback effect via fluid intelligence. The findings have implications for how we view vigilance as a cognitive construct, why it typically wanes with time, and how it may be stabilized. All data and materials are publicly available on the Open Science Framework (https://osf.io/fzd2h/)

**Supplementary Information:**

The online version contains supplementary material available at https://doi.org/10.3758/s13414-026-03314-8.

Historically, vigilance has been used to describe situations in which an observer must monitor a stream of signals for rare yet important target events. Some applied examples of vigilance include lifeguarding and transportation security. Embedded among minutes or even hours of nonsignals, target events (e.g., a drowning swimmer, a weapon in luggage) must be responded to quickly and appropriately. Laboratory paradigms have been developed to mimic such scenarios. Indeed, the original *vigilance decrement* was demonstrated using a simulated radar monitoring task (J. H. Mackworth, 1948). Despite the wealth of research, there is not yet consensus on why people experience time-on-task performance decrements, especially in situations that require a high degree of attention. For the purposes of the present study, we will refer to two general categories of theorizing: a) resource depletion and b) resource sharing.

Both the resource-depletion and resource-sharing accounts rest on the assumption that the human cognitive system is capacity limited. The basis of the resource-depletion account is that vigilance tasks are attentionally demanding, effortful, and importantly, they exhaust cognitive resources over time (Grier et al., 2003; Smit et al., 2004; Warm et al., 2008). Therefore, as time progresses, the pool of available resources shrinks, causing performance decrements to occur. These resources are presumed to be physiological in nature, but replenishable via rest. Evidence consistent with this account comes from studies showing steeper decrements to performance when the vigilance situation is more difficult (e.g., successive vs. simultaneous discriminations, lower signal-to-noise ratios; See et al., 1995). Further, people tend to report feeling stressed and fatigued after performing a vigilance task. Therefore, the resource depletion account argues that the exertion of effort toward the maintenance of vigilance is both physiologically and psychologically draining.

Resource-sharing accounts posit altogether different mechanisms. For example, the opportunity cost model of mental effort argues that attention-demanding tasks introduce a resource-sharing dilemma (Kurzban et al., 2013; Thomson et al., 2015). Since attention-demanding tasks consume one’s limited cognitive capacity, they introduce a necessary trade-off between maintaining high performance and attending to other concerns (e.g., other tasks, internal thoughts). Over time, one’s attention to the task may wane because the allocation policy shifts from full attention to a task to a sharing of resources between the primary task and attending to other things, like mind-wandering or reminiscing. Thus, according to the opportunity cost model (Kurzban et al., 2013), shifts in task performance with time are caused by a shift in attentional priorities. Thomson et al. (2015) offer a similar account with their resource-control model of sustained attention. They argue that the availability of cognitive resources does not change over time. Rather, as executive control wanes, the relative split of resources dedicated externally to the task and internally to mind-wandering shifts. The cost/benefit model is similar (Boksem & Tops, 2008). It argues that there are costs associated with maintaining a high level of attention, and these costs must be offset by benefits (e.g., rewards). When they are not, the system adaptively reduces the amount of effort applied, and therefore task performance declines. Finally, the motivational-control theory of cognitive fatigue proposes that people experience mental fatigue, and thus decrements to performance with time, when both a) the demands of the task are high and b) the individual does not feel a sense of agency or control over their activities (Hockey, 2011). For the purposes of the present study, we will cluster these theories into a resource-sharing group, since none of them implicate the depletion or exhaustion of a pool of physiological resources. Rather, these theories posit that attentional resources are split between the ongoing task and other forms of mentation. Evidence consistent with these accounts comes, for example, from studies showing improvements to vigilance when participants are offered performance-contingent rewards (e.g., Brewer et al., 2017; Esterman et al., 2014, 2016; Garner et al., 2023; Hopstaken et al., 2015a, 2015b, 2016; Massar et al., 2016, 2019; Robison & Nguyen, 2023; Strayer et al., 2023). We will contrast the predictions made by these theories, which are highly overlapping, against those made by the resource-depletion account.

Any investigation of an experimental manipulation designed to augment vigilance needs to consider at least two effects: a) a main effect of the manipulation on performance (i.e., a global change) and b) a time × manipulation interaction (i.e., a mitigation or elimination of the vigilance decrement). The presence of such effects, or lack thereof, can yield important insights into the phenomenology of vigilance and its associated time-on-task decrement. In general, the resource-depletion account predicts that any augmentation to a vigilance task that works to increase task engagement (e.g., via rewards, competition, performance feedback) should exert, at most, a main effect on task performance. According to resource theorists, the best way to improve or restore vigilance is with rest (Helton et al., 2015; Helton & Russel, 2017). With rest, the resources can be replenished. But even if an engagement-boosting manipulation produces a general improvement to vigilance, the effect could still be attributed to a physiological resource. For example, the “boost” could be due to an overall greater proportion of one’s available resource capacity being dedicated to the task. However, what is inconsistent with a resource account would be a significant mitigation, if not elimination, of the decrement in the absence of rest. That is, a manipulation that increases and stabilizes performance across time cannot be explained by resource depletion. Assuming the same amount of effort, or more, is exerted by the two conditions, the two conditions should nonetheless demonstrate similar time-on-task changes to performance. If there is mitigation/elimination of the decrement, the decrement must be due to the sustained management of cognitive resources, not their declining availability.

In the present study, we were specifically interested in the effects of periodic and summative performance feedback, often previously referred to at *knowledge of results*, on vigilance. Feedback presents a valuable manipulation which allows us to adjudicate between resource-depletion and resource-sharing theories. Additionally, feedback allows us to introduce the concept of metacognition as an explanatory mechanism for why feedback improves vigilance. As mentioned earlier, vigilance is often measured in paradigms that present rare targets among frequent nontargets. Further, targets and nontargets are typically difficult to distinguish, and in general people’s ability to distinguish between them wanes with time. That is, the vigilance decrement manifests as a decrease in sensitivity (i.e., distinguishing targets from nontargets) with time (see Thomson et al., 2016; McCarley & Yamini, 2021, for alternative explanations). Importantly, performance feedback improves vigilance in a variety of such paradigms (Becker et al., 1991; Chinn & Alluisi, 1964; Colquhoun et al., 1966; Grunzke et al., 1974; Loeb & Schmidt, 1963; J. F. Mackworth, 1964; Shaw et al., 2009; Sipowicz et al., 1962; Teo et al., 2012, 2013; Weidenfeller et al., 1962; Wiener, 1963; Wiener & Atwood, 1968). It appears to be a rather robust effect, but our review of the literature found several published instances in which feedback did *not* improve performance in a vigilance paradigm (Colquhoun et al., 1966; Helton et al., 1999; Szalma et al., 2000; Waldfogle et al., 2019).

Feedback-related performance benefits have also been observed in simple reaction time (RT) tasks, which tend to show robust decrements in the form of longer RTs as time-on-task progresses (Adams & Humes, 1963; Antonelli & Karas, 1967; Chylinski et al., 1962; McCormack et al., 1962; Robison et al., 2021; Warm et al., 1972, 1973, 1974). Interestingly, false feedback also has benefits (Antonelli & Karas, 1967; Mackworth, 1964; Weidenfeller et al., 1962). Under such conditions, participants believe the feedback is performance contingent, but it is delivered randomly or without reference to participants’ true performance. Finally, feedback manipulations have been applied to high “go” probability go/no-go tasks like the Sustained Attention to Response Task (SART), but with mixed results. For example, Mensen et al. (2022) alternately emphasized speed or accuracy and found that the feedback manipulations could affect one, but not both, aspects of performance. These findings were interpreted to mean that performance on such tasks reflects the speed–accuracy trade-off rather than vigilance or sustained attention per se. Similarly, Steinkrauss et al. (2023) intervened with feedback when participants’ RTs were particularly fast in the gradual-onset continuous performance task (gradCPT), which can produce errors. Doing so affected RTs, but it had little effect on error rates.

In some studies, performance feedback has only produced a main effect on performance (Adams & Humes, 1963; Robison et al., 2021; Warm et al., 1972). In these cases, resource-depletion can adequately explain the effect—the presence of feedback encourages people to engage with the task more fully, devoting more of their available cognitive capacity. However, conditions in which people do and do not receive feedback experience a similar decrement, and thus the depletion of resources may equally affect both conditions. But other times, feedback mitigates—or even eliminates—the vigilance decrement (Antonelli & Karas, 1967; Becker et al., 1991; Loeb & Schmidt, 1963; J. F. Mackworth, 1964; McCormack et al., 1962; Shaw et al., 2009; Sipowicz et al., 1962; Teo et al., 2013; Warm et al., 1973, 1974; Wiener, 1963, 1974). Resource depletion can explain the main effect of feedback on performance, but not its mitigation or elimination of a decrement.

Another interesting phenomenon is the finding that feedback during a vigilance task often has a transfer effect on performance in subsequent testing, even when the feedback has been removed (Adams & Humes, 1963; Teo et al., 2013; Warm et al., 1974; Wiener, 1963; Wiener & Attwood, 1968; but see Szalma et al., 2000, for a lack of transfer from feedback). For example, in a simple RT task, Warm et al. (1973) gave participants feedback whenever someone had emitted either their shortest reaction time, their longest reaction time, or no feedback. When all participants completed the task again during a testing phase, all without feedback, the participants who had been receiving feedback (either best or worst performance), outperformed those who had never received feedback. However, all groups showed a vigilance decrement in the testing phase. Teo et al. (2013) found improved vigilance for soldiers who received training with feedback on detecting improvised explosive devices (IEDs). On a later test, without feedback, they outperformed participants who did not receive feedback during training. However, a vigilance decrement remained.

To summarize, previous work has repeatedly found that providing performance feedback, under various forms, can improve vigilance globally, but also offset or even eliminate the classic vigilance decrement. However, in our review of the literature, we did not find an interpretation of feedback effects as evidence against a resource-depletion account of the decrement. But the simple provision of feedback creates a problem for resource depletion. Although it is plausible that receiving feedback might change an individual’s resource allocation policy, and therefore produce an increase in resources dedicated to the task, it is unclear how feedback would restore or otherwise offset the depletion of cognitive resources over time. Therefore, at most, the resource-depletion account would predict a main effect of feedback on vigilance, with those who do and do not receive feedback showing equal time-on-task decrements. Resource-sharing accounts, on the other hand, would predict that feedback would both increase performance overall and mitigate, or potentially eliminate, the decrement. Because resource-sharing accounts do not posit a reduction in the availability of resources over time, people can choose to maintain a stable level of resource allocation over time. These predicted patterns of data are shown in Fig. 1.

**Fig. 1:**
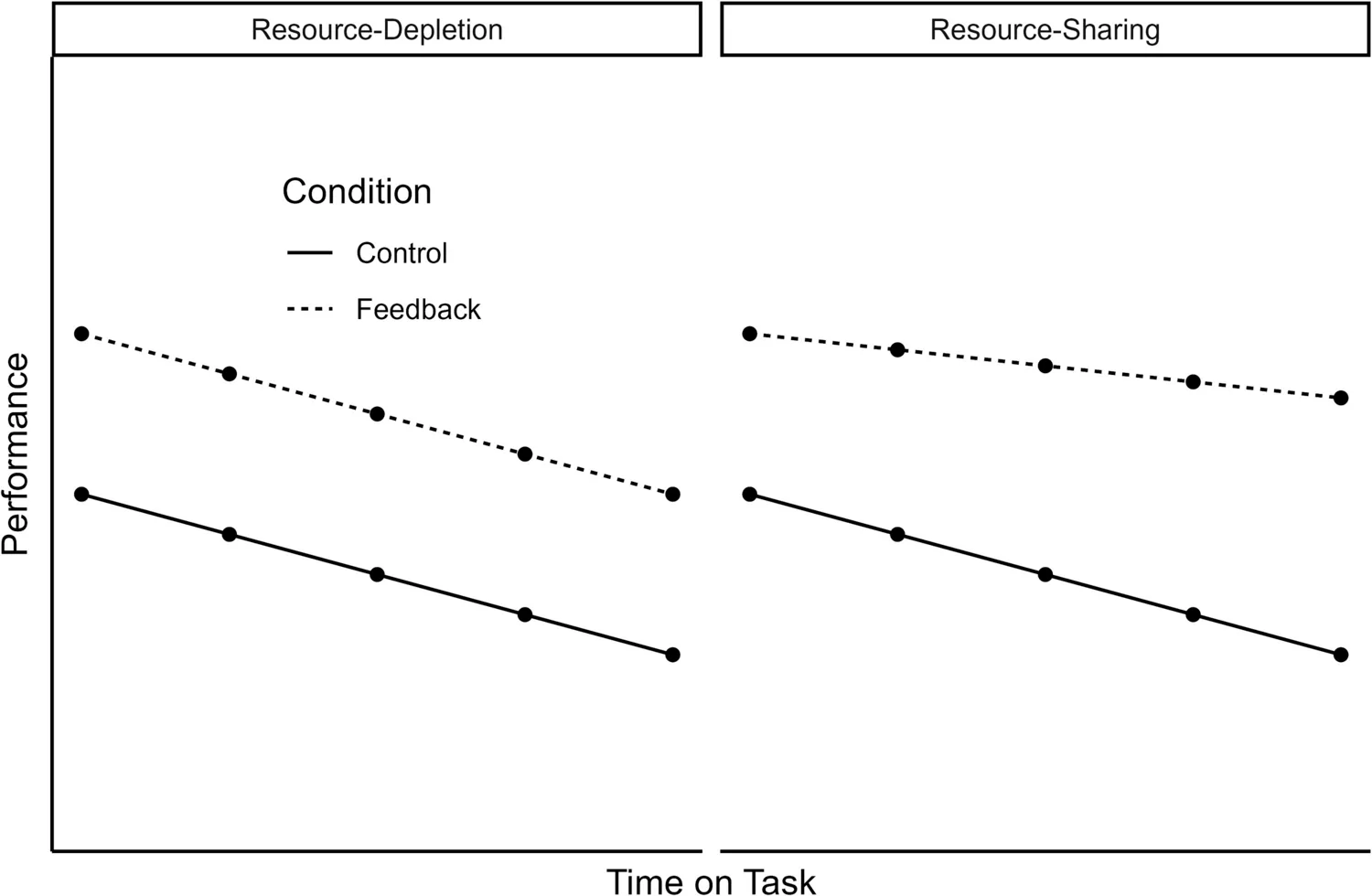
Predicted patterns of performance made by resource-depletion and resource-sharing theories

## Experiment 1

We used a common vigilance paradigm to test these predictions. At regular intervals, we inserted 10-s breaks between blocks. But in some conditions, we gave participants aggregate feedback about their performance in the prior block. In other conditions, participants did not receive such information.

## Method

### Participants and procedure

All participants were students at the University of Texas at Arlington (UTA) and Arizona State University (ASU) who completed a 2-hour laboratory session in exchange for partial course credit. After exclusions (see below), the final sample included 595 participants (*n =* 302 in feedback condition, *n* = 293 in control condition; 63% women, 34% men, 1% nonbinary, 2% did not report; *M*_age_ = 20.32 years, *SD* = 3.84). The sample size for Experiment 1 was preregistered for Campbell et al. (2026a) to estimate latent correlations of .50 to .80 within a corridor of stability of ±.10 with 80% confidence (minimum 274 per condition; Kretzschmar & Gignac, 2019). The achieved sample sizes are adequate to detect between-subjects effect sizes (Cohen’s *d*) of 0.30 with 95% power (α = .05, two-tailed). The procedure was administered in group run rooms with a maximum of eight participants per session. Participants first signed an informed consent document and completed a brief demographic survey. Then, all participants completed the following tasks in the same fixed order: color change detection, antisaccade, immediate free recall, Raven Advanced Progressive Matrices, orientation change detection, psychomotor vigilance, cued recall, abbreviated vigilance task (AVT), number series, Sustained Attention to Response Task (SART), letter change detection, picture source recognition, and letter sets. These data were collected as part of a larger combined individual-differences/experimental investigation of the effects feedback and motivation on the latent structure of cognitive ability (Campbell et al., 2026a). Here we focus specifically on the data from the abbreviated vigilance task (AVT). Later, we will also use data from the Raven, number series, and letter sets tasks for exploratory analyses.

Based on their subject ID number, participants were pseudorandomly assigned to either a feedback condition or a control condition (odd IDs = feedback, even IDs = control). In the feedback condition, participants received periodic feedback regarding their accuracy or reaction times, depending on the task. Then, at the conclusion of each task, participants were told their percentile score for that task using previously established norms. More details on the feedback for each task are provided below. The experimental protocol was approved by the Institutional Review Boards of UTA and ASU.

### Abbreviated Vigilance Task (AVT; Temple et al., 2000)

**Fig. 2:**
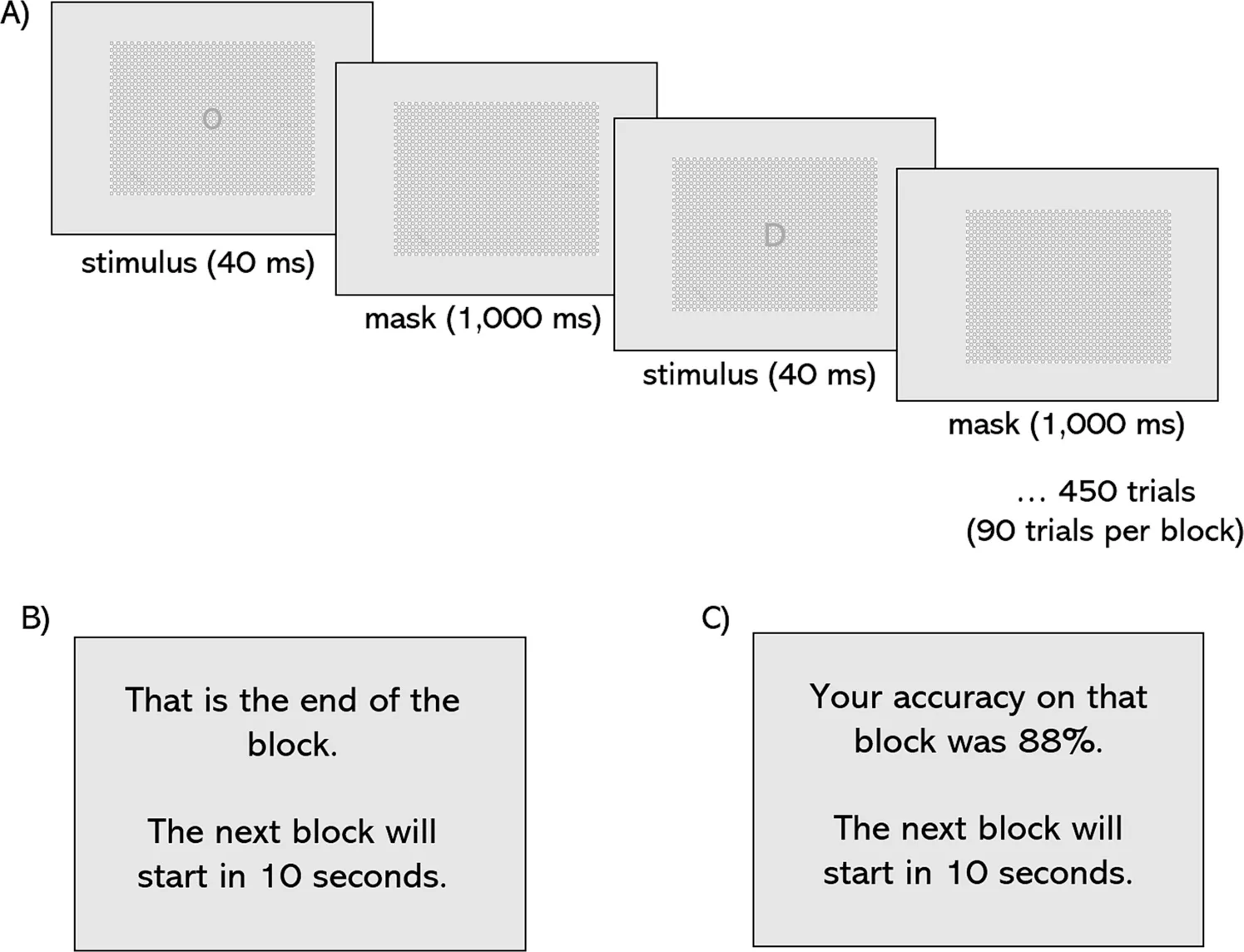
**A)** Trial sequence, **B)** control condition feedback, and **C)** feedback condition feedback. *Note.* Figure is for illustrative purposes and is not drawn precisely to scale

Participants completed 450 trials of the AVT, divided into five 90-trial blocks. Each trial of the AVT presented a gray character (0, *D*, or backward *D*) against a patterned background mask (see Fig. 2). The character flashed for 40 ms, followed by a 1,000-ms screen showing only the background mask (i.e., at a fixed 1,040-ms stimulus onset asynchrony). Participants were instructed to press the spacebar if and only if they saw a 0. They were to withhold any key press for Ds and backward Ds. Accuracy was stressed. There were exactly 15 target trials (i.e., 0s) and 75 nontargets (*D*s and backward *D*s) in each 90-trial block, presented in a different random order for each block and for each participant. We recorded both responses and response times. Responses were recorded as hits if a participant responded correctly to a target, as false alarms if a response was recorded to a nontarget, misses if a participant failed to response to a target, and correct rejections if no response was given to a nontarget. We then computed hit rates and false-alarm rates within each block for each participant. From these data, we computed a *d′* as the measure of sensitivity (*z*[hit rate] – *z*[false-alarm rate]; Hautus et al., 2022).^1^ There was no trial-by-trial feedback.

At the end of each block, participants in the feedback condition were shown a screen that said, “Your accuracy on that block was __%. The next block will start in __ seconds.” with a 10-s counter. The accuracy percentage for the preceding block was calculated as: (hit rate—false-alarm rate) × 100. In the control condition, participants were instead shown a screen that said, “That is the end of the block. The next block will start in __ seconds.” with the same 10-s countdown. At the end of the countdown, there was a 2-s blank screen before the task resumed. At the conclusion of the task, participants in the feedback condition were given their overall accuracy for the task and their position in a performance distribution with a percentile score. 2 In the control condition, participants were shown a screen that simply said, “That is the end of this task. Please get the experimenter.” There was one unanalyzed block of 48 practice trials at the beginning of the task. The duration of the task was 9 min.

### Motivation

After the practice trials and prior to Block 1, participants indicated their motivation to perform well on the task on a 9-point scale (1 = *not at all motivated*; 9 = *extremely motivated*).

### Analysis

The data were analyzed using R. Data were aggregated using the *data.table* (Dowle & Srivasan, 2021) package and the *tidyverse* (Wickham et al., 2019) suite of packages and plotted using the *ggplot2* (Wickham, 2016), *cowplot* (Wilke et al., 2019), and *ggrain* (Allen et al., 2021) packages. For the linear mixed effects models, we used the *lmerTest* package (Kuznetsova et al., 2017). To ensure the reproducibility of the analyses, we used the *groundhog* package (Simonsohn & Gruson, 2024) to tag each R package version to a date of March 1, 2025. Effect sizes for linear mixed models were estimated using the Satterwaithe approximation for degrees of freedom (*df*), the *t* value for the effect using the following formula: effect size (*d*) = (2 × *t*)/sqrt(*df*). All dependent variables were analyzed using a linear mixed effects model with fixed effects for condition (sum-to-zero effects coded with feedback = 1 and control = −1) and block (specified as a linear effect centered at 0). For all linear mixed effects models, we used the full random effects structure (random intercepts and random slopes; Barr et al., 2013). In some cases, a model with a random slope did not fit significantly better than a model with a random intercept only, indicating very little interindividual variation in the slope. However, to maintain consistency across experiments and ensure comparability in the analyses, we kept the random intercepts/random slopes structure throughout.

Although our primary measure of performance was sensitivity (*d′*), we also report analyses on hit rates, false-alarm rates, and response criterion (*c*). *C* was computed as −0.5 × [*z*hit rate + *z*false-alarm rate] (Hautus et al., 2022). If hit rates and/or false-alarm rates were exactly 1 or 0, they were corrected to be .99 or .01, respectively.^3^

### Exclusions

Per our preregistered data plan, participants were excluded if they had a greater false-alarm rate than hit rate in the AVT (*n* = 16 in control, *n* = 13 in feedback).^4^ Finally, we excluded data points that fell more than 3 standard deviations of their condition’s mean for a given block (0.30% of observations removed).

## Results

**Fig. 3:**
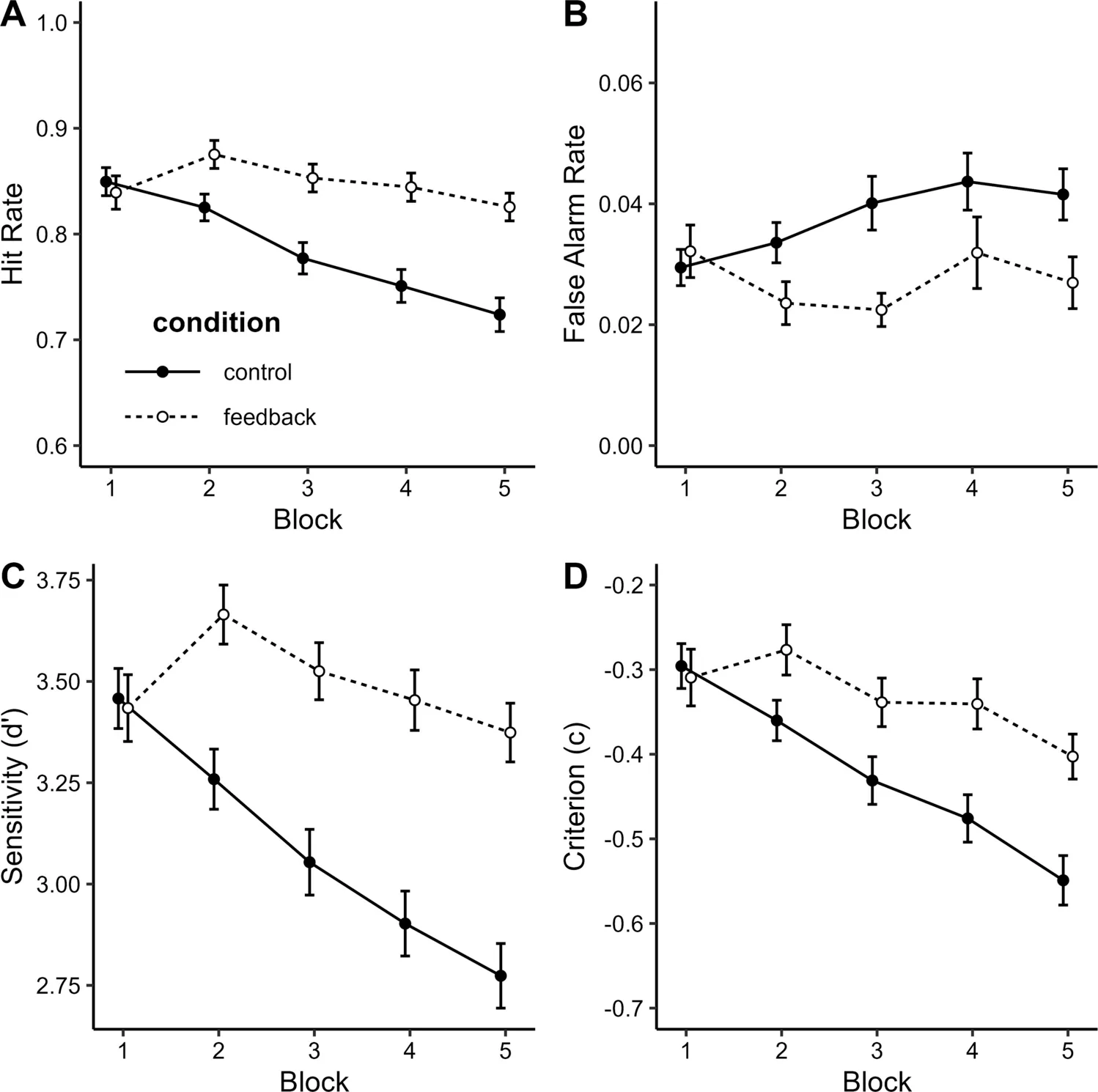
Task performance by block and condition in Experiment 1. Error bars represent ±1 standard error of the mean

Sensitivity (*d′*), hit rates, false-alarm rates, and response criterion (*c*) are plotted by block and condition in Figure 3.^5^ The model indicated significant main effects of block and block × feedback interactions on hit rates, false-alarm rates, and sensitivity (see Table 1). In summary, these effects indicate that participants in the feedback condition showed both better overall performance (i.e., higher hit rates, lower false-alarm rates, and thus higher sensitivity). To follow up on the block × feedback interaction on *d′*, we next specified separate models for the two conditions. There was a significant effect of block on sensitivity in the control condition (*b* = −0.17, *SE* = 0.02, *p* < .001, *d* = −1.43). The effect of block on sensitivity was much smaller in the feedback condition (*b* = −0.03, *SE* = 0.02, *p* = 0.05, *d* = −0.19). This is evidence that feedback significantly mitigated the vigilance decrement.

**Table 1:** Results of linear mixed models on dependent variables

Measure	Effect	*b*	*SE*	*p*	Effect size
*d′*	Block	−0.102	0.012	<.001	−0.722
	Feedback	0.197	0.048	<.001	0.339
	Block × Feedback	0.067	0.012	<.001	0.476
Hit rate	Block	−0.019	0.002	<.001	−0.647
	Feedback	0.031	0.009	<.001	0.294
	Block × Feedback	0.013	0.002	<.001	0.434
FA rate	Block	0.002	0.001	.058	0.157
	Feedback	−0.005	0.002	.044	−0.167
	Block × Feedback	−0.002	0.001	.026	−0.183
*c*	Block	−0.043	0.005	<.001	−0.674
	Feedback	0.045	0.016	.006	0.228
	Block × Feedback	0.018	0.005	.001	0.273

*d*′ = sensitivity measure, *FA* = false alarm, *c* = criterion. *b* = regression estimate, *SE* = standard error of estimate

A careful look at Fig. 3 reveals that the participants in the feedback condition showed a slight “bump” in performance after Block 1. Thus, the elimination of the decrement may have been driven by this change. To investigate this, we first computed within-person differences in sensitivity between Block 1 and Block 2 in each condition. Indeed, in the feedback condition, participants showed a significant increase, *t*(299) = 3.99, *p* < .001, *d*_z_ = 0.23 [0.12, 0.34], whereas in the control condition, they showed a significant decrease, *t*(290) = −3.68, *p* < .001, *d*_z_ = −0.22 [−0.33, −0.10]. Then, we estimated a linear mixed model ignoring Block 1. If this change from Block 1 to Block 2 were driving the effect, we should see an elimination of the block × feedback interaction when excluding Block 1 data from the model. However, this model still yielded a significant main effect of feedback (*b* = 0.25, *SE* = 0.05, *p* < .001, *d* = 0.41) and a significant block × feedback interaction (*b =* 0.03, *SE* = 0.01, *p* = 0.02, *d* = 0.19). It is worth noting that the effect size for the interaction dropped considerably from 0.48 to 0.19. So, the increase in sensitivity from Block 1 to Block 2 in the feedback condition accounted for some, but not all, of the mitigation of the vigilance decrement.^6^

As an alternative means of estimating the effect size of the vigilance decrement in each condition, we computed the effect size as the within-person difference in sensitivity between Block 1 and Block 5. In the control condition, the effect size was large (*d*_z_ = −0.60 [−0.72, −0.48]. However, in the feedback condition, the effect was essentially zero (*d*_z_ = 0.04 [−0.15, 0.07]. Comparing the magnitude of the vigilance decrements across conditions yielded a large difference (*d* = −0.52 [−0.68, −0.35].^7^ However, it is worth noting that the elimination was likely driven by the increase in performance from Block 1 to Block 2 in the feedback condition. Indeed, computing the magnitude of the decrement by subtracting Block 5 from Block 2 yielded a significant effect in the control condition (*d*_z_ = −0.50 [−0.62, −0.38] and in the feedback condition (*d*_z_ = −0.27 [−0.38, −0.15]). The difference in magnitudes was also significant (*d*_z_ = −0.19 [−0.35, −0.03]).

Finally, we also analyzed the self-reports of motivation and compared them across conditions. However, they did not differ between the feedback and control conditions (*feedback*: *M* = 5.56, *SD* = 2.54; *control*: *M* = 5.66, *SD* = 2.42), *t*(595) = 0.46, *p* = .64, *d* = 0.04 [−0.12, 0.20].

## Experiment 2

Experiment 1 demonstrated that feedback can mitigate the vigilance decrement in a difficult perceptual discrimination task. In Experiment 2, we were interested in whether the effect would remain for a longer period, in addition to directly replicating the findings from Experiment 1. As mentioned earlier, the participants in Experiment 1 completed the AVT within a larger task battery, and the participants in the feedback condition had been receiving both individualized and normative comparison feedback. Therefore, one effect of the feedback could have been the normative comparisons they had been receiving after tasks completed prior to the AVT. So, replication of the effect was warranted. In addition, we wanted to explore whether the same findings would hold for a longer task. To that end, we doubled the duration of the AVT to 900 trials (10 blocks) and retained the feedback manipulation.

## Method

We report how we determined our sample size, all data exclusions, all manipulations, and all measures in the study.

### Participants and procedure

A sample of 105 participants from the human subject pool at UTA completed the study in exchange for partial course credit. The sample size was preregistered based on a power analysis using G*Power (Faul et al., 2007) from an independent-samples *t* test of the difference in the magnitude of the vigilance decrement between the feedback and control conditions in Experiment 1 (*d* = 0.66, α = .05, one-tailed, 1 − β = .95). This analysis yielded a required sample size of 102 participants (51 per condition).^8^ After exclusions, we finished with a sample size of 99 (*n* = 50 in control condition, *n* = 49 in feedback; 78% women; 18% men, 4% did not report; *M*_age_ years = 18.89, *SD* = 1.56). The task was completed during a 1-hour laboratory session. During the first half of the session, participants completed a separate task that was not relevant to the current study. Participants were randomly assigned to various conditions in that task, as well, and therefore any carry-over effects were evenly distributed between the conditions in the vigilance task (see Garner et al., 2025). None of the participants had participated in Experiment 1.

### Abbreviated vigilance task

The task duration was doubled from five blocks of 90 trials each to 10 blocks of 90 trials each (18 min). Otherwise, the task was identical.

### Data analysis

We used the same analysis approach as Experiment 1. We excluded participants who had a higher false-alarm rate than hit rate and any participants who fell outside ±3 standard deviations of the mean.

## Results

**Fig. 4:**
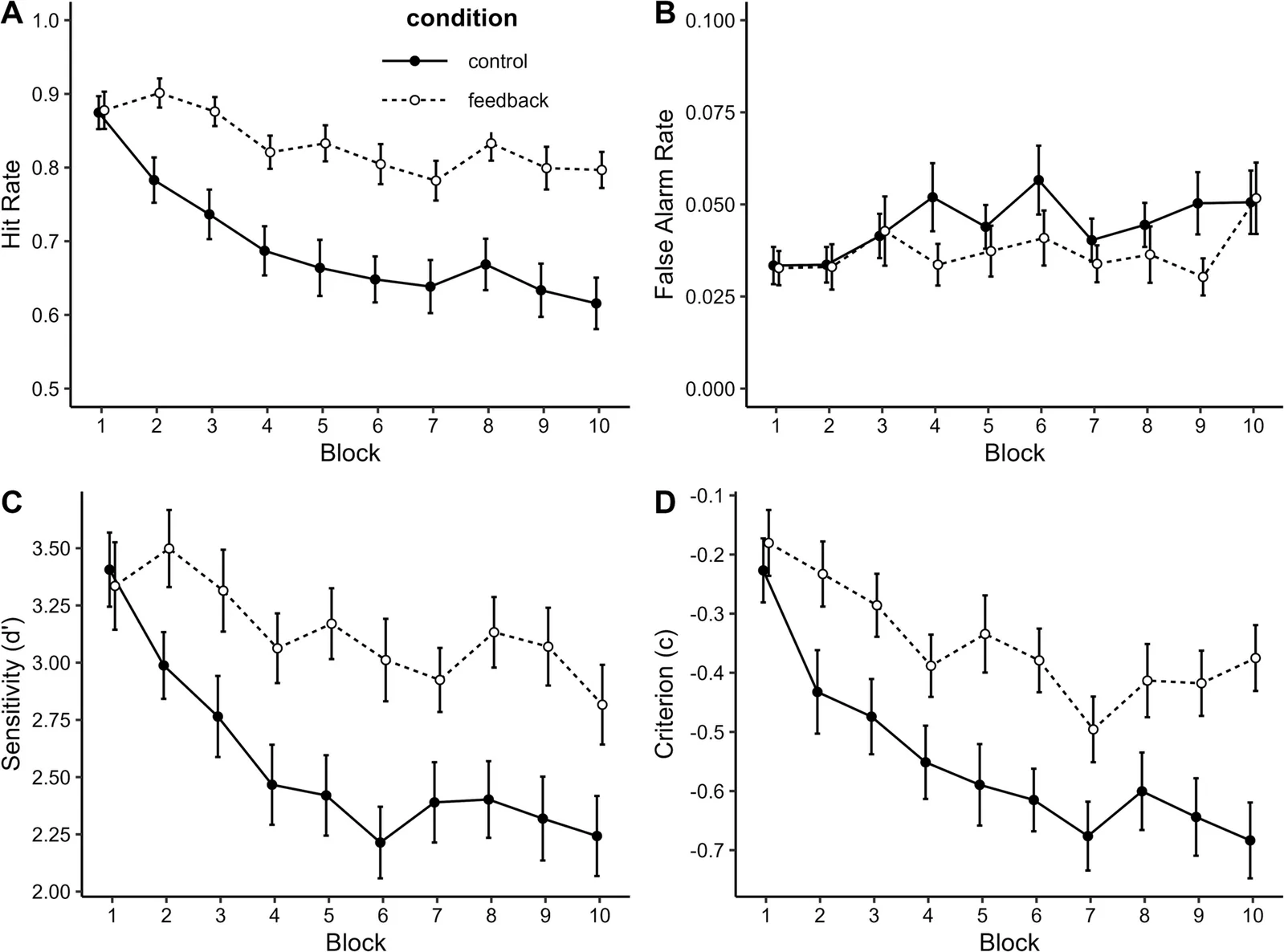
Task performance by block and condition in Experiment 2. Error bars represent ±1 standard error of the mean

Hit rates, false-alarm rates, sensitivity (*d′*), and criterion (*c*) are plotted by block and condition in Fig. 4. Table 2 includes summaries of the linear mixed effects models. The results were largely consistent with Experiment 1. Participants in the feedback condition showed significantly higher sensitivity overall and significantly shallower vigilance decrements, a sustained level of hits, and a less of a shift toward a more conservative response bias compared with the control condition. To decompose the block × feedback interaction on *d′*, we estimated the average vigilance decrement in each condition by comparing Block 10 to Block 1. In the control condition, the change in sensitivity was large (*d*_z_ = −1.09 [−1.45, −0.74]). The effect was also significant in the feedback condition, albeit smaller (*d*_z_* = −*0.40 [−0.70, −0.10]), with difference in differences being large (*d* = −0.80 [−1.22, −0.38]).

**Table 2:** Summary of linear mixed models on dependent variables in Experiment 2

**Measure**	**Effect**	***b***	***SE***	***p***	**Effect size**
*d′*	Block	−0.089	0.011	<.001	−1.648
	Feedback	0.273	0.105	.011	0.537
	Block × Feedback	0.033	0.011	.004	0.606
Hit rate	Block	−0.017	0.002	<.001	−1.547
	Feedback	0.067	0.018	<.001	0.777
	Block × Feedback	0.009	0.002	<.001	0.812
FA Rate	Block	0.004	0.002	.022	0.691
	Feedback	0.002	0.010	.823	0.046
	Block × Feedback	0.001	0.002	.718	0.106
*c*	Block	−0.029	0.005	<.001	−1.299
	Feedback	0.111	0.032	<.001	0.716
	Block × Feedback	0.012	0.005	.033	0.529

* d′* = sensitivity measure, FA = false alarm, *c* = criterion. *b* = regression estimate, *SE* = standard error of estimate.

Unlike Experiment 1, participants in the feedback condition did not show a significant increase in *d′* from Block 1 to Block 2, *t*(42) = 0.85, *p* = .40, *d*_z_ = 0.13 [−0.17, 0.42]. However, in the control condition, there was a significant decrease in sensitivity from Block 1 to Block 2, *t*(48) = −3.94, *p* < .001, *d*_z_ = −0.55 [−0.85, −0.26]. When removing Block 1 from the linear mixed model on *d′*, there was still a significant main effect of feedback (*b* = 0.29, *SE* = 0.11, *p* = .007, *d* = 0.56) and a significant block × feedback interaction (*b* = 0.03, *SE* = 0.01, *p* = .004, *d* = 0.60).^9^ Therefore, the immediate change from Block 1 to Block 2 did not entirely account for the difference in vigilance decrements between conditions.

Similar to Experiment 1, there was no significant difference in pre-task motivation ratings in the feedback (*M* = 5.96, *SD* = 1.90) and control conditions (*M* = 6.34, *SD* = 2.10; *t*(97) = 0.94, *p* = .35, *d* = 0.19 [−0.21, 0.58).

## Experiment 3

Experiments 1 and 2 produced nearly identical patterns of results. Periodic performance feedback eliminated the vigilance decrement in both conditions. The results were more consistent with a resource-sharing account of vigilance. The central question, then, is how does receiving performance feedback eliminate the vigilance decrement? There are several plausible explanations.

The first potential mechanism is feedback as an extrinsic motivator. Perhaps the presence of objective performance feedback encourages people to devote more attention to the task because seeing high performance scores after each block is motivating (Kluger & DeNisi, 1996; Warm et al., 1972, 1974). Here, we did not observe any differences between conditions in pretask self-reports of motivation. This is not surprising because the ratings were made before participants started the task and not mid task or post task. Therefore, we could not adequately assess whether motivation was a driving factor here. However, as mentioned earlier, there is evidence that receiving feedback has a lasting impact on vigilance, even after it has been removed (Adams & Humes, 1963; McCormack, 1959; Teo et al., 2012, 2013; Wiener, 1974). This finding casts doubt on the primary mechanism of feedback being an extrinsic motivator. If the feedback serves primarily as a reward, its removal should also remove its effect on performance. But this is not always observed. The fact that having previously received feedback—even on an entirely different day—produces a carry-over effect on performance challenges the notion that the effects of feedback are due mostly to changes in motivation.

Another way for feedback to assist vigilance is through a metacognitive scaffolding mechanism. This hypothesis is compared to a motivational account in Experiment 3. According to a metacognitive mechanism, vigilance decrements occur at least in part because people may not be metacognitively aware of their own deteriorations in performance. Therefore, their performance may continue to slip without their realizing it. Or people may notice their errors, but because their performance is never reported back to them, the errors are less salient. In either case, the objective feedback provides an external support to the metacognitive system. Kluger and DeNisi’s (1996) process model proposes that feedback allows for an objective comparison between a person’s current performance level and their desired performance level, creating a feedback-standard discrepancy. If the discrepancy is positive (performance > standard), people can either increase their effort to attain even higher performance, or they can maintain the current level of effort. If the discrepancy is negative (performance < standard), people can increase their effort. If that upward adjustment in effort subsequently improves their performance, they can maintain or increase effort further. But if the increased effort does *not* improve performance, people may update their beliefs about whether the standard is an achievable goal. In so doing, the feedback may be providing direct input to the metacognitive monitoring process and allowing for better adjustments of metacognitive control. It is providing a salient external cue for the regulation of behavior.

**Fig. 5:**
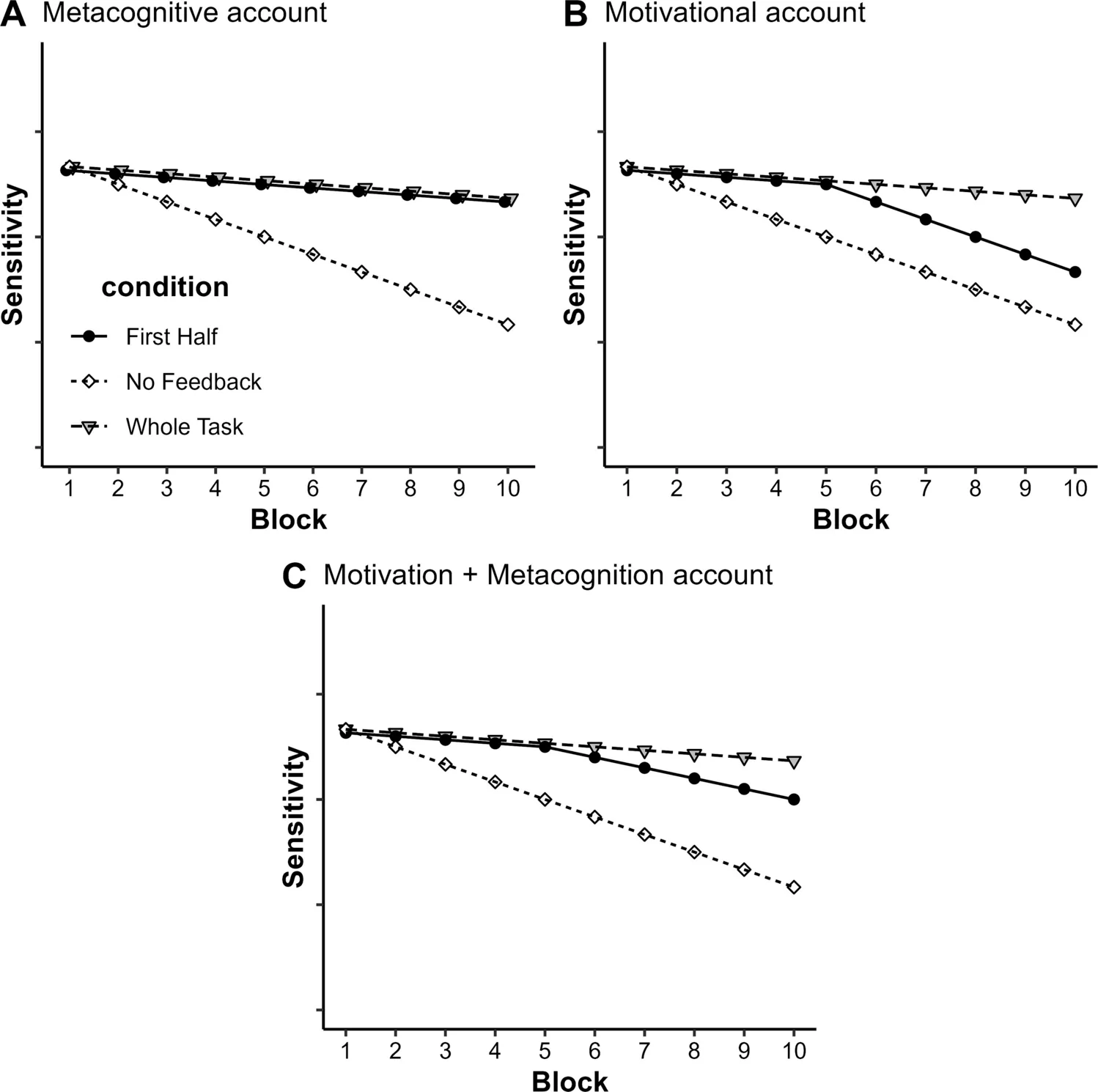
Predictions made by a metacognitive, motivational, or combined effect of feedback

In Experiment 3, we administered three conditions of the AVT: one in which participants received feedback for the entire 10-block duration (whole-task condition); one in which participants received feedback for the first five blocks, but not for the last five blocks (first-half condition); and one in which participants did not receive any feedback (no-feedback condition). We also included a self-report question regarding task-based motivation prior to Block 1, after Block 5, and after Block 10. We hypothesized that if the effect of feedback is primarily metacognitive in nature, such that it helps people calibrate their own metacognitive monitoring and implement better metacognitive control processes, providing feedback should provide a lasting advantage to participants who have received it, even after it has been removed. Therefore, participants in the first-half condition should continue to exhibit superior vigilance to participants in the no-feedback condition even after it has been removed. However, if feedback is primarily motivational in nature, participants in the first-half condition should show similar vigilance as participants in the no-feedback condition, and worse than participants in the whole-task condition, after the feedback has been removed. Further, motivation after Block 5 should be lower in the no-feedback condition compared to the other two conditions, and at the conclusion of the task, participants in the first-half condition should show similar motivation to the no-feedback condition. It is also possible that both metacognition *and* motivation influence performance. If this is the case, participants who in the first-half condition will have a steeper vigilance decrement in Blocks 6 through 10 than the whole-task condition, but significantly shallower vigilance decrement than participants in the no-feedback condition in Blocks 6 through 10. These hypotheses were preregistered on OSF (https://osf.io/bwk4y). They are visualized in Fig. 5.

## Method

### Participants and procedure

Participants were all undergraduate students (*M*_age_ = 19.34 years, *SD* = 1.09; 68% women, 32% men) from the human subject pool at the University of Notre Dame.^10^ The target sample size was 78 participants per condition (total *N* = 234) based on a power analysis conducted in G*Power (Faul et al., 2007) indicating a minimum sample size necessary for a between-subjects effect size of 0.40, one-tailed, α = .05, power = .80. The effect of interest was the block × feedback interaction. We stopped collecting data at the mid-semester break of the Spring 2025 semester. After exclusions, the final sample included 281 participants (*n* = 107 in the first-half condition, *n* = 87 in whole-task condition, and *n* = 87 in no-feedback condition). Participants completed the study as part of a 2-hour task battery. The other tasks were measures of cognitive abilities not intended to be part of this investigation; however, we report on data from the Raven, number series, and letter sets tasks. Participants read and signed an informed consent document and completed a brief demographic survey before starting any tasks. The experimental protocol was approved by the Institutional Review Board at the University of Notre Dame.

#### AVT

The task design and duration (900 trials divided into 10 blocks lasting a total of 18 min) were nearly identical to Experiment 2. We added three self-report motivation ratings. One occurred after the practice trials and prior to Block 1; one occurred between Blocks 5 and 6, and one occurred after Block 10. The self-report scales asked participants to, “Please rate how motivated you feel to perform well on the task.” on a 9-point scale (1 = *not at all motivated*; 9 = *extremely motivated*).

### Raven Advanced Progressive Matrices (Raven et al., 1962)

On each trial, participants saw a 3 × 3 grid of geometrical patterns. The bottom-right piece of the grid was missing. Based on an implicit set of rules, participants were to select from a set of eight possible options the piece that best completed the grid. Participants completed the 18 odd-numbered items. The items increased in difficulty as they progressed. There was a 10-min time limit to complete as many items as possible. The dependent variable was the total number of correct responses (max score = 18).

### Number series (Thurstone, 1938)

Participants were given a set of numbers and asked to select from a set of five possible options which number best continued an implicit pattern. Participants were given 4.5 min to complete as many items as possible. There were 15 items total. All participants received the same items in the same order. The dependent variable was the total number of correct responses (max score = 15).

### Letter sets (Ekstrom et al., 1976)

Participants were given five sets of four letters (e.g., ABAA CDCC XYXX HGHH) and asked to select the set that did not match the pattern present in the other three sets. Participants were given 5 min to complete as many items as possible. There were 15 items total. All participants received the same items in the same order. The dependent variable was the total number of correct responses (max score = 15).

### Analyses

To examine the effects statistically, we estimated two sets of linear mixed-effects models. In the first set, we examined only performance in the first half of the task (Blocks 1 through 5). For these analyses, the effect of feedback was sum-to-zero effects coded with condition = 1 for the first-half and whole-task conditions and −1 for the no-feedback condition. In the second set of analyses, we examined only performance in the second half of the task (Blocks 6 through 10). For these analyses, the effect of feedback was sum-to-zero effects coded with condition = 1 for whole-task conditions and −1 for the no-feedback and first-half conditions.

### Exclusions

Participants were excluded based on a preregistered set of criteria. We first excluded participants whose false-alarm rates were greater than or equal to their hit rates (*n* = 2 in the whole-task condition, *n* = 2 in no-feedback condition). We then used a *z*-score threshold of ±3 over each condition, eliminating participants who fell outside this range for *d′* within a block within a condition. We deviated from our preregistration indicating that we would exclude any participants with fewer than five blocks of included data. We instead excluded participants with fewer than eight blocks of included data. This procedure eliminated 26 participants in the first-half condition,^11^ two participants in the whole-task condition, and two participants in the no-feedback condition.

## Results

**Fig. 6:**
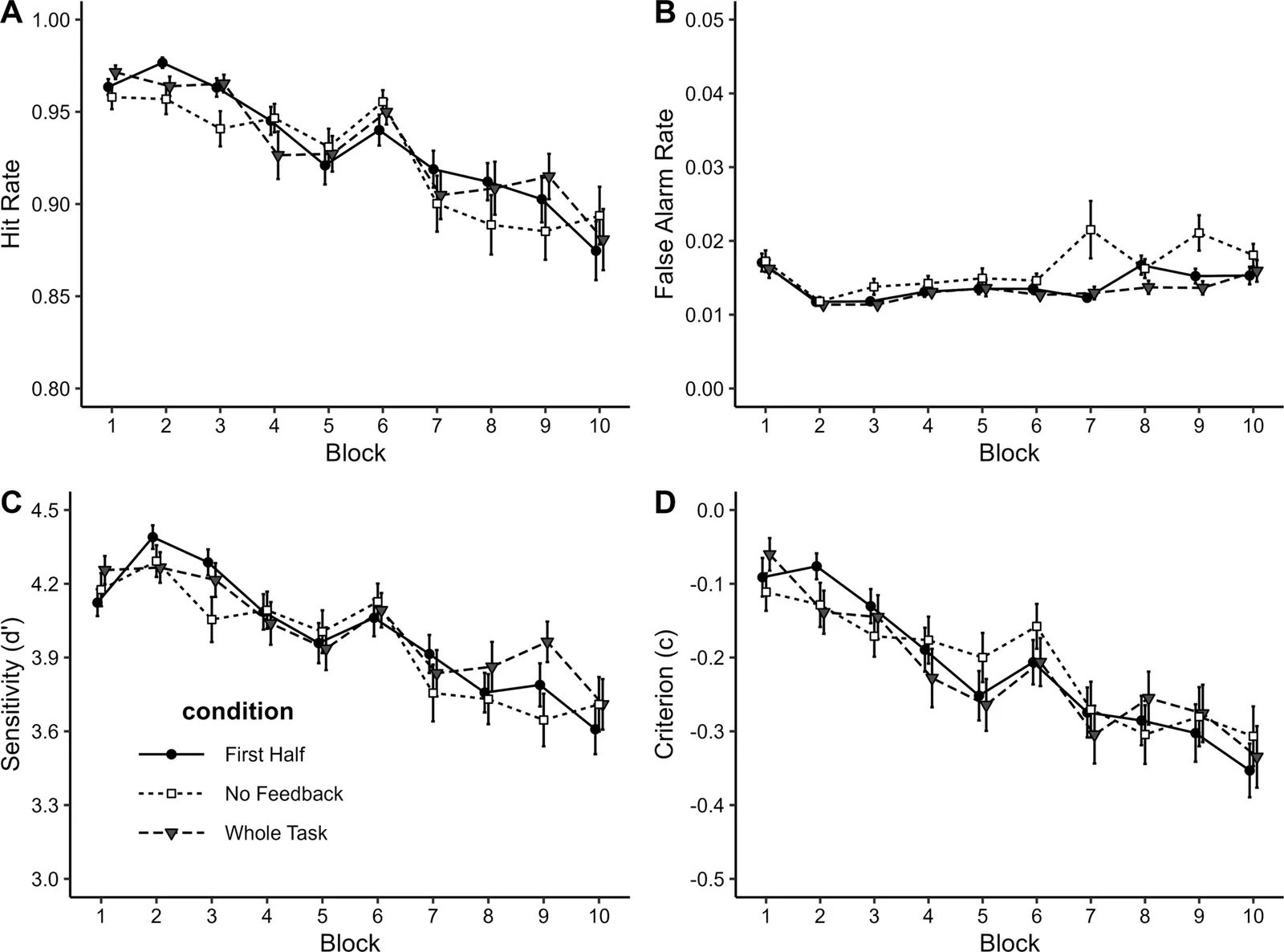
Task performance by block and condition in Experiment 3. Error bars represent ±1 standard error of the mean

The data are plotted by block and condition in Fig. 6. As can be seen in the figure, there was very little separation between the conditions. The first set of models (Blocks 1 through 5) are summarized in Table 3. Although there was a significant vigilance decrement, the effect did not differ between conditions, and there were not significant overall performance differences in any of the four variables.

**Table 3:** Results of linear mixed models for Experiment 3 (first five blocks)

Measure	Effect	*b*	*SE*	*p*	Effect size
*d′*	Block	−0.054	0.012	<.001	−0.533
	Feedback	0.026	0.035	.472	0.089
	Block × Feedback	−0.013	0.012	.279	−0.133
Hit rate	Block	−0.011	0.002	<.001	−0.677
	Feedback	0.003	0.005	.531	0.078
	Block × Feedback	−0.003	0.002	.072	−0.222
FA rate	Block	−0.001	0.000	.173	−0.166
	Feedback	−0.001	0.001	.366	−0.114
	Block × Feedback	−0.0002	0.0004	.557	−0.071
*c*	Block	−0.036	0.006	<.001	−0.716
	Feedback	0.003	0.012	.786	0.033
	Block × Feedback	−0.010	0.006	.092	−0.205

*d′* = sensitivity measure, *FA* = false alarm, *c* = criterion. *b* = regression estimate, *SE* = standard error of estimate

The second set of models (Blocks 6 through 10) indicated largely the same pattern (see Table 4). Collectively, the three conditions showed significant decreases in hit rates, increases in false-alarm rates, decreases in sensitivity, and a shift toward a conservative response criterion. However, there were no significant differences between the conditions, either overall or in the magnitude of the effects with time.

**Table 4:** Results of linear mixed models for Experiment 3 (last five blocks)

Measure	Effect	*b*	*SE*	*p*	Effect size
*d′*	Block	−0.081	0.013	<.001	−0.742
	Feedback	0.043	0.049	.380	0.107
	Block × Feedback	0.016	0.013	0.245	0.143
Hit rate	Block	−0.014	0.002	<.001	−0.697
	Feedback	0.004	0.008	.602	0.064
	Block × Feedback	0.003	0.002	.261	0.139
FA rate	Block	0.0003	0.001	.807	0.041
	Feedback	−0.003	0.002	.111	−0.206
	Block × Feedback	0.001	0.001	.511	0.111
*c*	Block	−0.028	0.006	<.001	−0.374
	Feedback	−0.002	0.018	.902	−0.015
	Block × Feedback	0.007	0.006	.235	0.093

*d′* = sensitivity measure, *FA* = false alarm, *c* = criterion. *b* = regression estimate, *SE* = standard error of  estimate

**Fig. 7:**
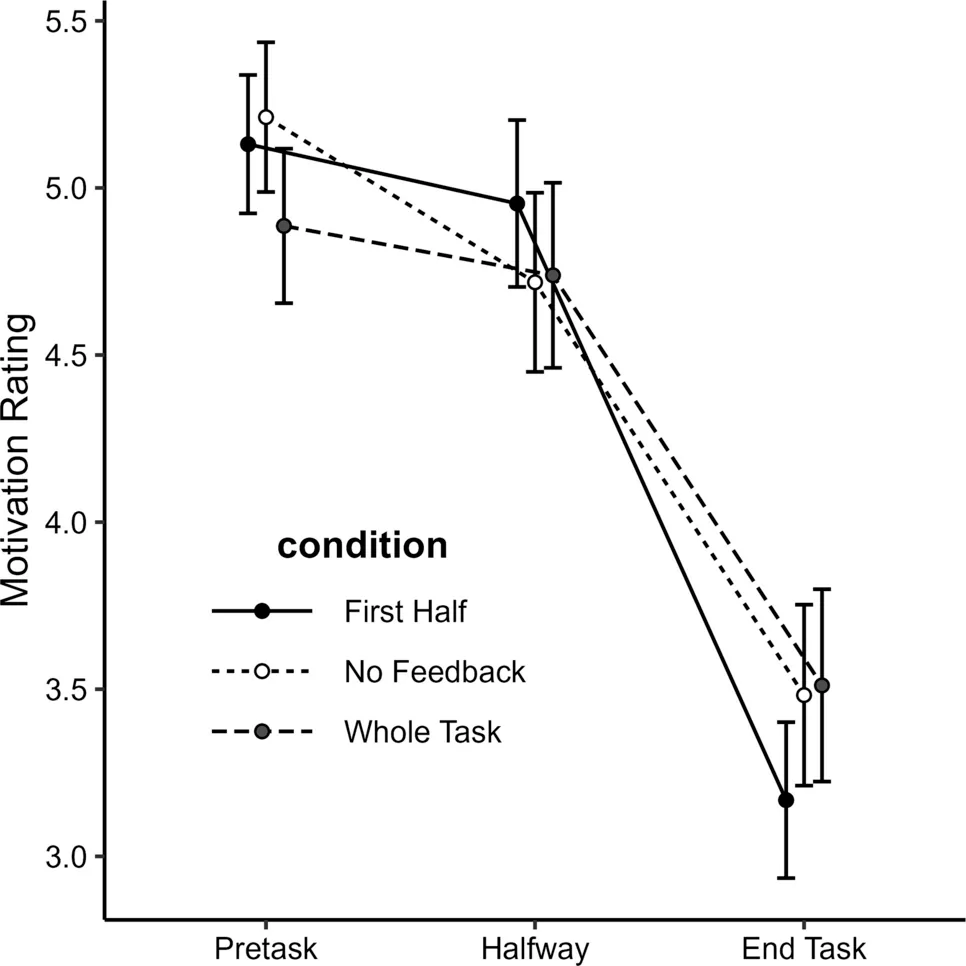
Motivation ratings by time and condition in Experiment 3. Error bars represent ±1 standard error of the mean

The motivation ratings are plotted by condition and time-on-task in Fig. 7. We analyzed these ratings with a 3 (condition) × 3 (time) mixed ANOVA. The ANOVA indicated a significant main effect of time-on-task, *F*(2, 554) = 99.27, *p* < .001, η_p_^2^ = .26, but no significant main effect of condition, *F*(2, 277) = .04, *p* = .96, η_p_^2^ < .001, and no significant time × condition interaction, *F*(4, 554) = 1.58, *p* = .18, η_p_^2^ = .01. The time to make the motivation ratings did not differ between conditions, *F*(2, 275) = 0.15, *p* = .86, η_p_^2^ = .001), and although participants took less time to make their halfway and end-task ratings compared with the pretask ratings, *F*(1.26, 347.67) = 47.15, *p* < .001, η_p_^2^ = .146, this effect did not differ interact with condition, *F*(2.53, 347.67) = 1.12, *p* = .34, η_p_^2^ = .008). Participants made their motivation ratings in an average of 5.44 seconds (*SD* = 3.25), mitigating any concerns that participants used this stopping point in the task as a “break.”

### Combined analyses

Because there was no effect of feedback on task performance, none of our preregistered hypotheses (i.e., motivational account vs. metacognitive account vs. combined account) were supported by the data. Although all three conditions showed a significant vigilance decrement, a decrease in sensitivity, a decrease in hit rates, and a shift toward a more conservative response criterion, we did not see a significant difference in either overall performance or the magnitude the vigilance decrement across conditions. Further, although all three conditions reported significantly lower motivation at the end of Block 10 than they did prior to Block 1 and at the end of Block 5, there were no differences in overall motivation or changes in motivation across conditions.

**Fig. 8:**
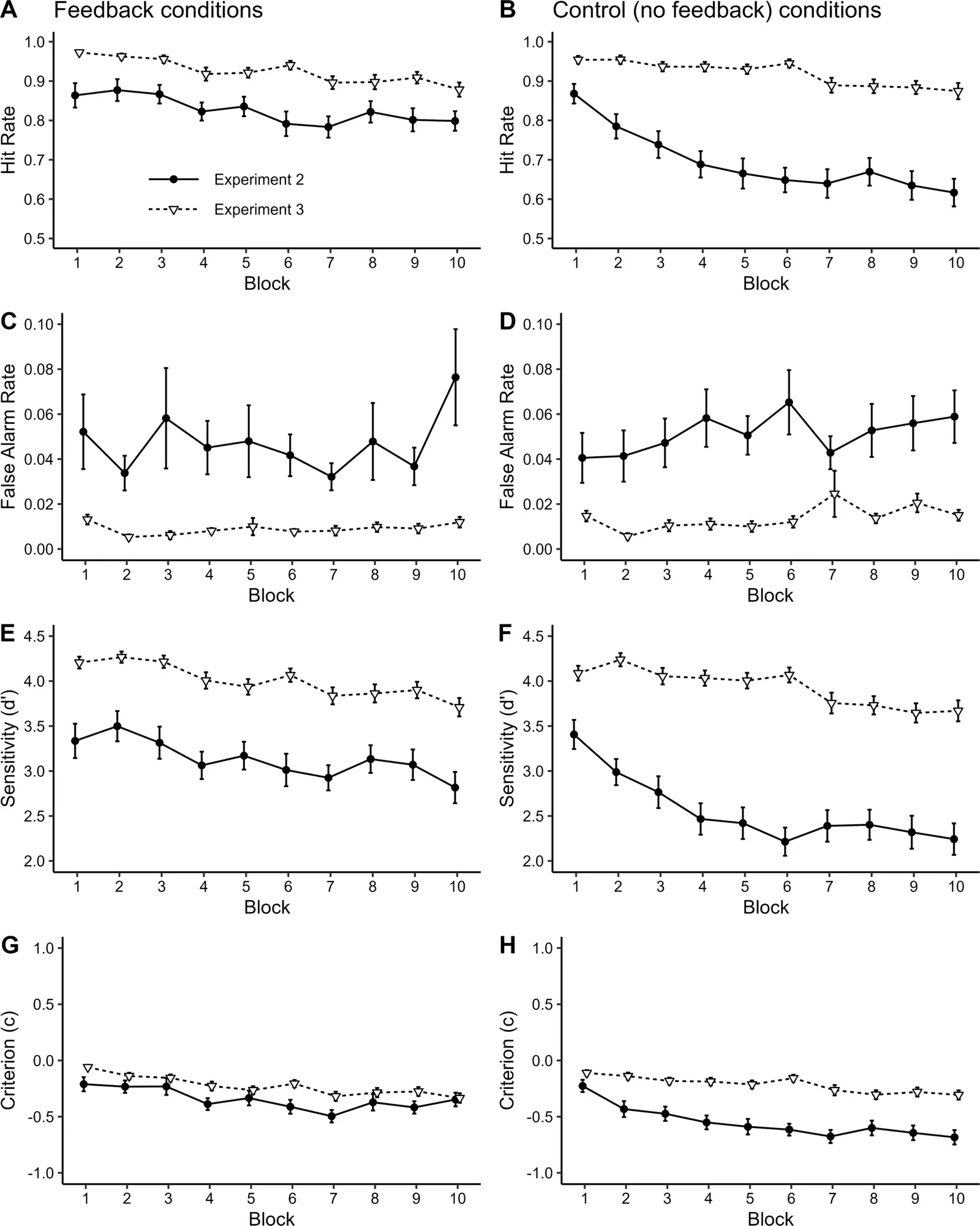
Task performance comparisons of Experiments 2 and 3. Error bars represent ±1 standard error

This pattern of results thus begs the question: why did our feedback manipulation, which was robust in both Experiments 1 and 2, not have any effect in Experiment 3? The tasks were nearly identical in Experiments 2 and 3, with the exception of the inclusion of the first-half condition and the three motivation self-reports. Therefore, our first step in comparing the samples was to compare the whole-task and no-feedback conditions of Experiment 3 to the feedback and control conditions of Experiment 2, which were largely the same. Those comparisons are plotted in Fig. 8. As is visible, participants in Experiment 3 significantly outperformed participants in Experiment 2. Comparing performance in the two feedback conditions revealed that participants in the whole-task condition in Experiment 3 significantly outperformed participants in the comparable feedback condition in Experiment 2 by having higher overall hit rates, lower false-alarm rates, higher sensitivity, but also a more *liberal* response criterion.

**Fig. 9:**
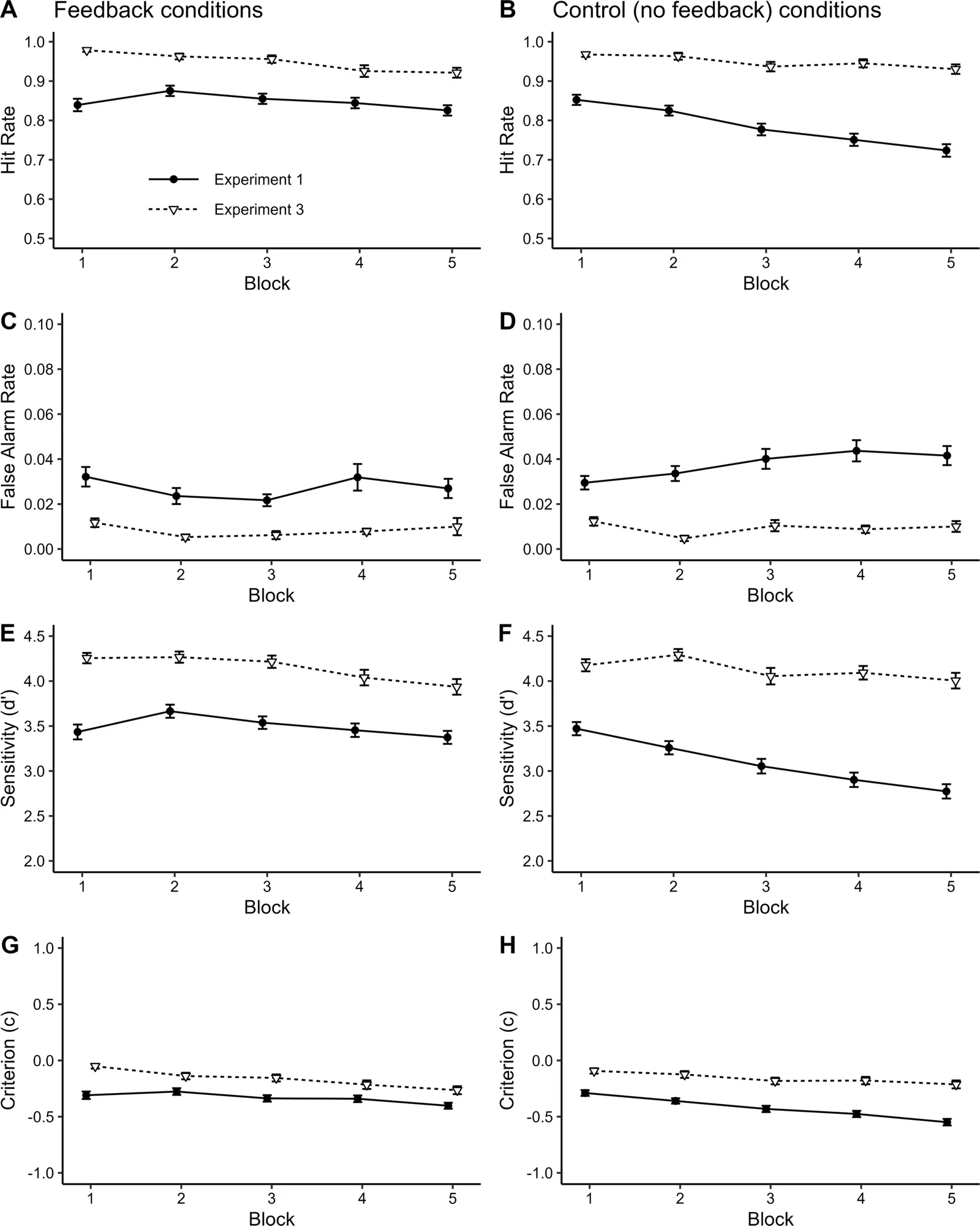
Task performance comparisons of Experiments 1 and 3. Error bars represent ±1 standard error

Another fair comparison is between performance in the first five blocks in the combination of the whole-task and first-half conditions of Experiment 3 and the feedback condition of Experiment 1. Those comparisons are plotted in Fig. 9. Again, participants in Experiment 3 had higher sensitivity, higher hit rates, and lower false-alarm rates.

These comparisons indicate that there were significant differences in performance and the magnitude of the feedback effect between the sampling locations. However, this does not yield much insight into *why* these differences arose. Although we had not planned on incorporating the data into the analysis, we happened to use three identical measures of fluid intelligence (Raven, number series, and letter sets) in the two experiments as part of larger studies with different goals (Campbell, et al., 2026a, b). We surmised that one possibility for the elimination of the effect of feedback might have been driven by the sample compositions. Participants in Experiments 1 and 2 were all students at the University of Texas at Arlington (UTA) and Arizona State University (ASU), and the participants in Experiment 3 were all students at the University of Notre Dame. Whereas Notre Dame is a highly selective university, which admits only 12% of its annual undergraduate applicant pool (U.S. News & World Report, n.d.-b), UTA’s admissions rate is about 81% (U.S. News & World Report, n.d.-c) and ASU’s is about 90% (U.S. News & World Report, n.d.-a). Therefore, samples at Notre Dame will tend to be much more exclusive and potentially range restricted in terms of cognitive ability. Thus, one possible explanation is the highly restricted sample from which our data were drawn in Experiment 3.

**Fig. 10:**
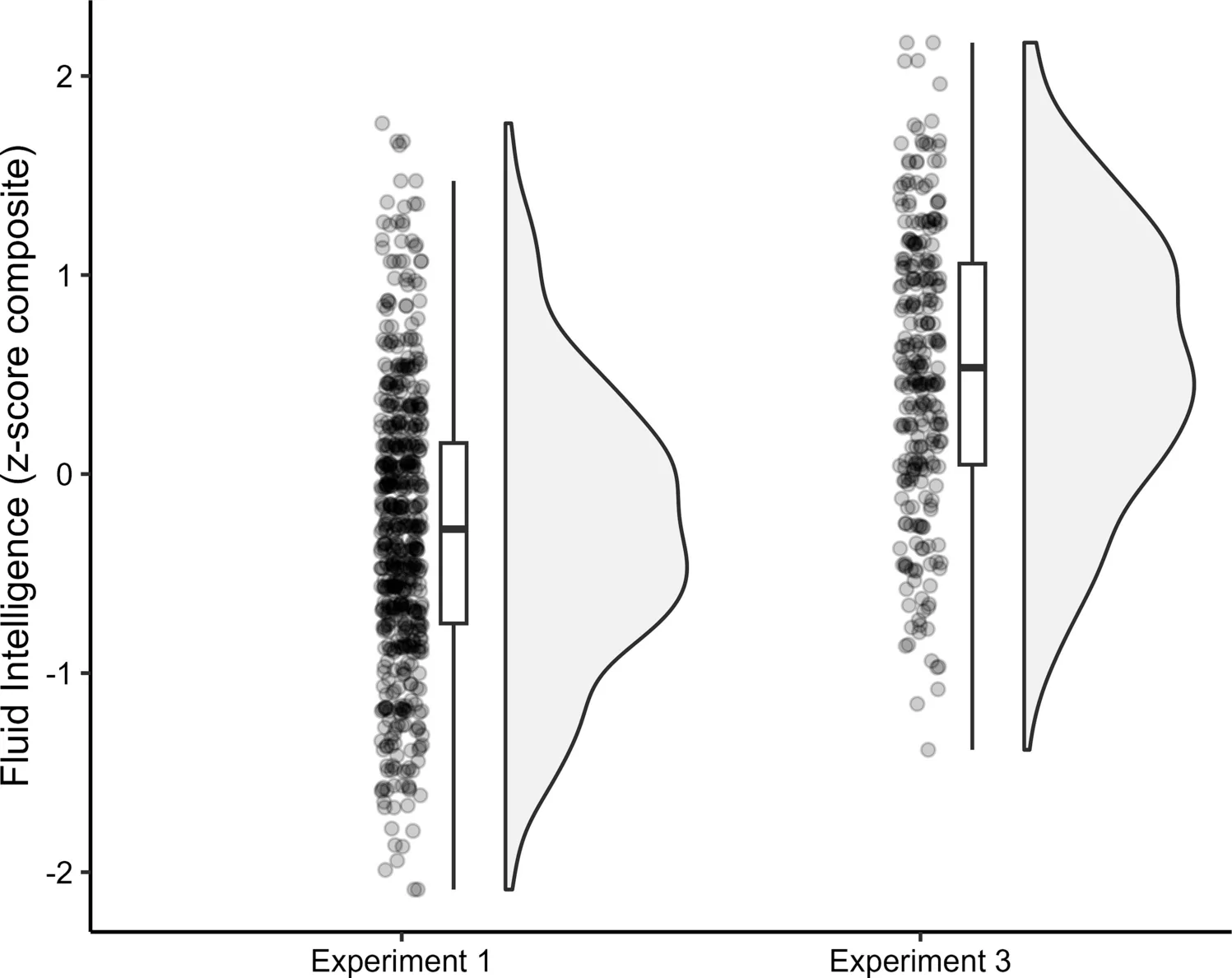
Distributions of fluid intelligence (*z*-score composites) in Experiments 1 and 3. Each dot represents a single participant. The boxplots and density plots show the distributions in each experiment

Using the Raven, number series, and letter sets tasks, all of which have been used previously to measure a latent fluid intelligence ability (Burgoyne et al., 2023; Draheim et al., 2021; Robison & Campbell, 2023; Robison et al., 2024; Robison et al., 2026), we can more specifically test whether a discrepancy in the range and distribution of fluid intelligence between the two samples could have accounted for the washout of the feedback effect in Experiment 3. First, we compared average performance on the Raven, number series, and letter sets tasks between Experiments 1 and 3. These comparisons are listed in Table 5. On average, participants in Experiment 3 had significantly higher performance on the Raven, number series, and letter sets tasks, and the effect sizes were all large. Given that these three tasks were all included to measure one construct, and that they were all significantly correlated,^12^ we also computed a *z*-score composite by averaging standardized scores on the three tasks. The *z*-score composite comparison also yielded a large difference in average fluid intelligence performance (see Fig. 10). Interestingly, the standard deviations in the two samples were almost identical. Thus, although there was certainly an overall difference in the average fluid intelligence in the two samples, there was an equal amount of variability. Therefore, the range did not appear to be more restricted in the Notre Dame student sample compared to the UTA and ASU student samples.

**Table 5:** Fluid intelligence task performance comparisons

Measure	Experiment 1 (*n* = 586)		Experiment 3 (*n* = 278)		Comparison		
	Mean	*SD*	Mean	*SD*	*t*	*p*	Effect size
Letter sets	7.86	2.74	11.25	3.34	15.45	<.001	1.11
Number series	7.58	2.90	10.33	2.67	13.28	<.001	0.99
Raven	8.54	3.35	10.20	3.20	6.83	<.001	0.51
*Z-*score composite	−0.28	0.71	0.53	0.71	15.64	<.001	1.14

*SD* = standard deviation

Given the large mean differences and relatively high performance, the lack of an effect of feedback in Experiment 3 might be driven by cognitive ability differences across samples. Therefore, we conducted another set of models in which we included a fixed effect for fluid intelligence in addition to fixed effects for block and feedback and all possible interactions. These models allow us to ask a theoretically meaningful question: Do individual differences in fluid intelligence moderate the effect of feedback on the vigilance decrement? If so, we should observe a three-way interaction among the effects of block, feedback, and fluid intelligence. Indeed, that is exactly what we observed. Table 6 lists model summaries. There were significant block × fluid intelligence × feedback interactions on sensitivity, hit rate, and false-alarm rate. Importantly, this interaction was not present for *c*, indicating that the effects were not due to differences in response criterion.

**Table 6:** Results of linear mixed models examining moderating effect of fluid intelligence

**Measure**	**Effect**	***b***	***SE***	***p***	**Effect size**
*d′*	Block	−0.092	0.009	<.001	−0.714
	Fluid Intelligence	0.184	0.027	<.001	0.326
	Feedback	0.653	0.032	<.001	0.896
	Block × Fluid Intelligence	0.048	0.009	<.001	0.345
	Block × Feedback	0.010	0.011	.385	0.055
	Fluid Intelligence × Feedback	−0.156	0.035	<.001	−0.223
	Block × Fluid Intelligence × Feedback	−0.031	0.011	0.007	−0.177
Hit rate	Block	−0.017	0.002	<.001	−0.690
	Fluid Intelligence	0.031	0.005	<.001	0.301
	Feedback	0.096	0.006	<.001	0.734
	Block × Fluid Intelligence	0.009	0.002	<.001	0.326
	Block × Feedback	0.003	0.002	.117	0.098
	Fluid Intelligence × Feedback	−0.022	0.006	<.001	−0.174
	Block × Fluid Intelligence × Feedback	−0.007	0.002	0.002	−0.197
FA rate	Block	0.001	0.001	.240	0.083
	Fluid Intelligence	−0.006	0.001	<.001	−0.240
	Feedback	−0.021	0.002	<.001	−0.670
	Block × Fluid Intelligence	−0.002	0.001	<.001	−0.227
	Block × Feedback	−0.001	0.001	.303	−0.064
	Fluid Intelligence × Feedback	0.005	0.002	.001	0.182
	Block × Fluid Intelligence × Feedback	0.001	0.001	.024	0.146
*c*	Block	−0.044	0.004	<.001	−0.767
	Fluid Intelligence	0.043	0.010	<.001	0.224
	Feedback	0.143	0.013	< .001	0.577
	Block × Fluid Intelligence	0.010	0.004	.012	0.165
	Block × Feedback	0.001	0.005	.884	0.009
	Fluid Intelligence × Feedback	−0.021	0.013	.112	−0.088
	Block × Fluid Intelligence × Feedback	−0.004	0.005	.418	−0.053

*Note. d′* = sensitivity measure, FA = false alarm, *c* = criterion. *b* = regression estimate, *SE* = standard error of estimate.

**Fig. 11:**
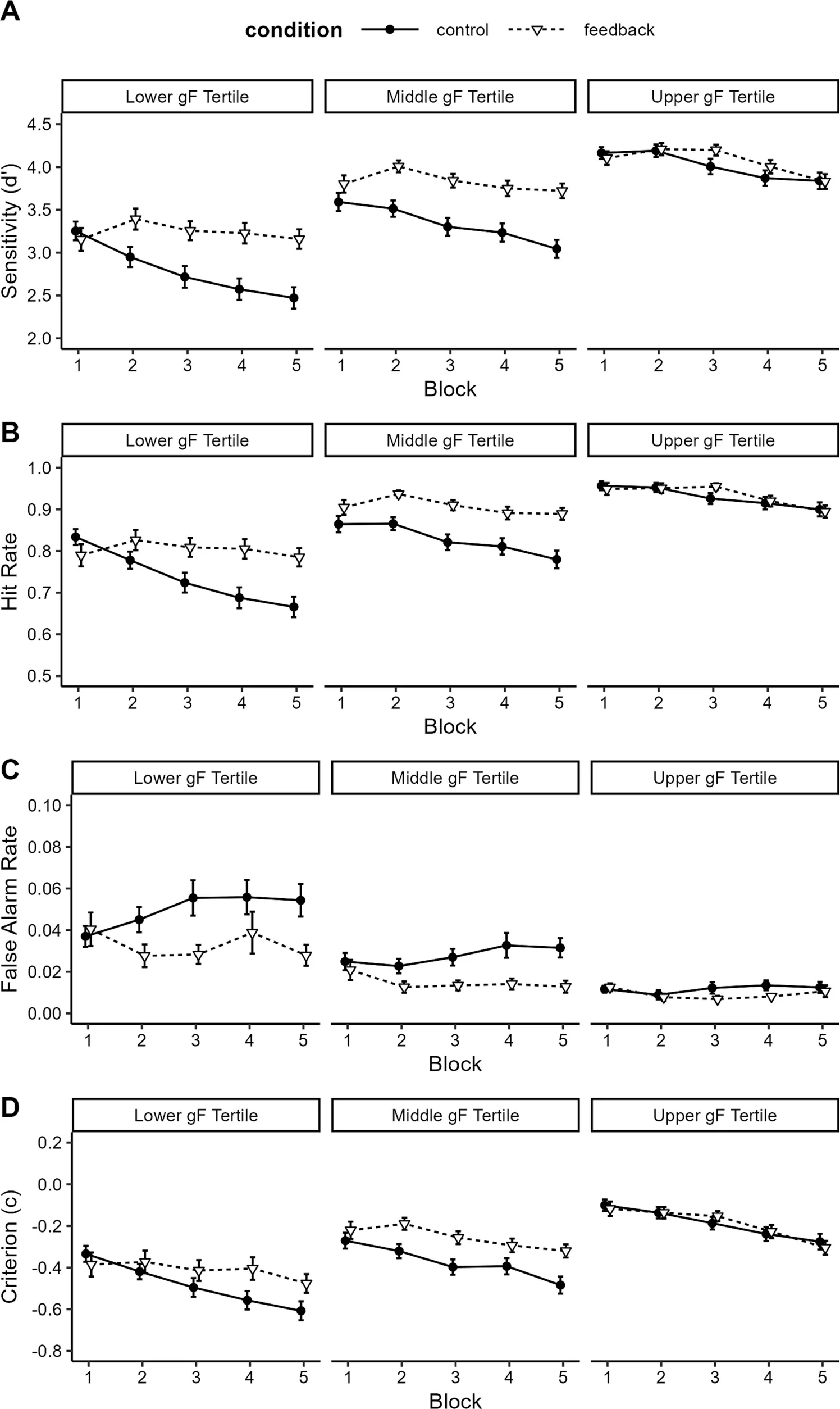
Moderation of feedback effect by fluid intelligence. Error bars represent ±1 standard error of the mean. gF = fluid intelligence

The moderation is plotted in Fig. 11. As can be seen, the effect of feedback was largest among participants on the lowest end of the intelligence distribution and was smallest at the highest end of the distribution. That is, even without feedback, the highest-ability participants were performing the task well enough that the provision of feedback did not improve their performance compared to their fellow high-ability participants. However, at the lower end of the ability distribution, feedback had large effects. This adds a caveat on our findings: The effects of feedback are not uniformly distributed along the distribution of individual differences in cognitive ability. It was most effective for those of the lowest fluid intelligence from whom we sampled, and virtually ineffective among those with the highest fluid intelligence in the sample.

## General discussion

In three experiments we examined the effect of periodic performance feedback on vigilance. In Experiments 1 and 2 there was robust mitigation of the vigilance decrement with feedback. We interpreted these findings to indicate that vigilance decrements are likely not driven by resource depletion and more likely due to resource-sharing. That is, the results can be explained as a difference in the cognitive resources allocated to the task across time based on whether or not one is receiving feedback about their performance. Since feedback might work to increase allocation to the task, it reduces the magnitude of the decrement. In Experiment 3, we attempted to replicate this phenomenon and add a third condition to adjudicate between two potential mechanisms for feedback’s elimination the decrement: motivation and metacognition. However, because we observed no effects of feedback in Experiment 3, we could not fully adjudicate between them. The fact that the conditions did not report different levels of motivation, either before, during, or after the task, would qualify as evidence against a motivational account. However, we also did not find immediate evidence in favor of the metacognitive account due to the lack of performance differences among conditions.

The elimination of the between-subject effect of feedback in Experiment 3 led us to perform some additional exploratory analyses. Specifically, we wondered whether sampling from a highly selective undergraduate sample may have hindered our ability to observe an effect of feedback in Experiment 3. Indeed, relative to Experiment 1, the participants in Experiment 3 scored significantly higher on measures of fluid intelligence. Incorporating these scores into the analyses indicated a significant moderation of the feedback effect by fluid intelligence. This brought an unexpected but important caveat to our findings: Feedback was particularly effective for people of relatively lower cognitive abilities, in this case fluid intelligence. Given this finding, do the results have any bearing on why feedback does (and sometimes does not) affect vigilance? Does it influence how we think about vigilance more generally? There are at least a few possibilities.

The first possibility is a metacognitive mechanism, despite the lack of support for this hypothesis in Experiment 3. Metacognition has knowledge, monitoring, and control components (Dunlosky & Metcalfe, 2008). The knowledge components are facts or beliefs people know about their own cognition, such as the fact that reading a book and listening to a podcast are difficult to do at the same time. The monitoring components are the processes by which people ascertain whether their ongoing thoughts and cognitions have been successful or not. Finally, control components are those processes by which people dynamically adjust their thoughts and behaviors in response to the output of the monitoring process. Performance feedback may therefore constitute salient *external* support, and therefore they would not be considered part of metacognition. But instead, feedback can assist and improve the effectiveness of the metacognitive system should it go awry.

Specifically, there are at least two ways in which feedback improves vigilance via metacognition. As opposed to a depletion of resources (Grier et al., 2003; Smit et al., 2004; Warm et al., 2008), Thomson et al. (2015) explain changes in performance as a changing allocation policy which is governed by executive control. Control changes over time, they argue, either because of an adaptation to the low-frequency target event rates of vigilance task, or because the effort associated with maintaining a high level of control is costly (Kurzban et al., 2013). Therefore, because allocating attention is effortful, there will be a persistent pull toward a state of rest (i.e., a lower attentional allocation strategy), and participants will satisfice. That is, they will use the minimum amount of effort required to obtain an acceptable level of performance. This pull toward rest can be counteracted by either internal or external support systems. Metacognition is one such internal support system. Metacognitive monitoring allows one to “keep score” of one’s performance accurately such that adjustments of attentional allocation (i.e., metacognitive control) can be implemented. Those internal systems are imperfect yet can be supported by external features of the environment, such as feedback. When provided, the external and internal systems thus work together to counteract the pull toward rest and maintain a higher level of attentional allocation. People by nature derive satisfaction from performing well, and therefore will be motivated to reduce a performance discrepancy or otherwise maximize performance (Kluger & DeNisi, 1996). Likewise, recognition that performance has declined, via either internal metacognitive monitoring or external feedback, is aversive, and such recognition will generate a response (i.e., a reallocation of attention).

The significant moderation of the feedback effect by fluid intelligence could indicate that one aspect of having higher fluid intelligence is superior metacognitive monitoring and control systems (Hertzog & Robinson, 2005; Murphy et al., 2021; Saraç et al., 2014; Schunn & Reder, 2001). To highlight some relevant examples, participants who are higher in fluid intelligence have shown better metacognitive monitoring and control in a value-directed remembering paradigm (Murphy et al., 2021); students with higher fluid intelligence showed better metacognitive monitoring in a text-learning task (Saraç et al., 2014); and Schunn and Reder (2001) found that inductive reasoning ability, measured similarly to fluid intelligence in the present study, is a predictor of strategic adaptivity in a simulated air traffic control task. Finally, Hertzog and Robinson (2005) report that inductive reasoning, which is conceptualized similarly to fluid intelligence, is among the strongest predictors of strategic memory use in associative learning tasks. Collectively, these studies demonstrate that people who tend to score higher in fluid intelligence tend to use metacognitive processes to a better extent. Therefore, feedback may have been more effective for the participants lower in fluid intelligence because it provided more external support for their metacognitive monitoring and control abilities. For the high fluid intelligence participants, these metacognitive monitoring and control processes may have already been operating at a high level, even in the absence of feedback.

A metacognitive mechanism has been implicated in vigilance in previous work. Maniscalo et al. (2017) argue that a lack of feedback requires one rely on their own metacognitive monitoring system, rather than that process being offloaded to an external feedback system. However, their interpretation is much different from ours. The metacognitive process, they argue, requires cognitive resources and thus magnifies resource depletion. Thus, it is a marriage of metacognitive and a resource-depletion accounts. However, this account is inconsistent with prior work which has asked people to make self-judgments of performance periodically, thus heightening the need for participants to metacognitively monitor. According to this account, self-reporting one’s own performance should sharpen vigilance decrements. However, self-reporting performance has been shown to *reduce* vigilance decrements similarly to objective feedback (Warm et al., 1973).

Alternatively, both the main effect of fluid intelligence and its interaction with the feedback manipulation could also be due to the high degree of overlap between fluid intelligence and related cognitive abilities like working memory capacity and executive-attention. There is a well-established connection between individual differences in fluid intelligence and individual differences in attention control (Burgoyne et al., 2023; Draheim et al., 2021; Engle et al., 1999; Robison et al., 2024; Robison et al., 2026; Tsukahara et al., 2020; Unsworth et al., 2014; Unsworth & Spillers, 2010), and it has previously been shown that people with higher working memory capacity tend to perform better on measures of sustained attention (Unsworth & Robison, 2020). In the present study, performance (*d′*) was also significantly correlated with individual differences in our fluid intelligence composite (Experiment 1: *r* = .25 [.17, .31], Experiment 3: *r* = .19 [.09, .30]). Therefore, it might be that fluid intelligence influences vigilance via their shared relation with working memory and/or domain-general executive-attention ability. One critical aspect of executive-attention is goal maintenance (Engle & Kane, 2004), and therefore the feedback might work to reactivate and strengthen the maintenance of the task goal across time, particularly for people with lower fluid intelligence.

A third potential mechanism is the motivating influence of feedback. The results of Experiment 3 are most informative here. Even after experiencing five and then ten blocks of the task with and without feedback, participants did not differ in self-reported motivation. If the feedback were highly motivating, we should have seen greater motivation reported by participants in the first-half and whole-task conditions compared with the no-feedback condition. Further, once feedback had been removed, participants in the first-half condition should have showed a steeper drop in motivation from the end of Block 5 to the end of Block 10 compared with the other conditions. It is worth noting that we do not believe motivation is not an important element of vigilance. Indeed, self-reported motivation was significantly correlated with *d′* in all three experiments (Experiment 1: *r* = .24 [.16, .31], *p* < .001; Experiment 2: *r* = .43 [28, .58], *p* < .001; Experiment 3: *r* = .30 [.20, .40], *p* < .001). However, the mechanism explaining feedback’s effect on vigilance does not appear to be driven by motivation as the primary mechanism. Warm et al. (1973) make a similar case when they say “the motivational effects of [knowledge of results] may reside predominantly in its ability to enhance the self-evaluative activity associated with it” (p. 292).

Fourth, a resource-depletion account could argue that decrements to performance only occur after resources have been depleted to a certain level, and what the feedback is doing is keeping the *allocation* of resources above that level, even if they are still being depleted. A future test of this hypothesis would be to examine whether feedback has differential effects on the decrement in tasks that vary in their demands and thus should theoretically deplete resources faster. Resource-depletion theories might also argue that there is room in depletion theories for resources to be replenished even over brief intervals (Helton & Wen, 2023). However, it is unclear why participants in feedback conditions would experience more replenishing of resources during brief self-imposed or experimenter-imposed rest periods than participants in the no-feedback conditions. Finally, it might be argued that feedback offsets the vigilance decrement not via increased metacognitive monitoring or control activities but through an increase in the efficiency with which the task is being completed. This account would argue that the number of resources required by the task decreases with time due to increased efficiency, and therefore resource depletion occurs at an ever-decreasing rate. While plausible, we would argue this account is unlikely because it requires the difficult perceptual discriminations required in the task to become automatized over a short (~10 to 20 min) period of time. However, this could also be tested in the future by gradually increasing the difficulty of the task (e.g., signal intensity, successive vs. simultaneous comparison) and compare it to conditions under which difficulty remains constant, with and without feedback. All of these theories, of course, can also be criticized for their incorporation of a resource concept in the first place, as resources have been criticized previously for being too vague a notion to be theoretically testable (Allport, 1993; Navon, 1984; Neumann, 1987).

Finally, there has been debate regarding the precise reasons for performance changes as a function of time in vigilance tasks. Thomson et al. (2016) argue that changes in performance across time are driven mostly by a shift toward a conservative response bias, not by changes in sensitivity. This point has been disputed by Fraulini et al. (2017). To follow up on this debate, McCarley and Yamani (2021) conducted an experiment and used Bayesian hierarchical modeling to examine whether changes in performance with time were associated with a shift in response bias, a change in sensitivity, or an increase in lapse rate. While they found evidence for changes in all three parameters, the effect was mostly driven by a shift in response bias (i.e., criterion). We could not directly replicate the analyses of McCarley and Yamani (2021) here because we did not manipulate signal intensity, which was a critical feature of their design. However, it is worth noting that we observed significant shifts in response bias (*c*) in our experiments that were sometimes as large or larger than changes in sensitivity (*d′*). Further, in Experiments 1 and 2, where we observed significant mitigation of the change in sensitivity with time by feedback, we also observed significant mitigation of the changes in response bias with time by feedback. Therefore, our results are consistent with McCarley and Yamani (2021) that changes in vigilance may be caused by multiple underlying factors, two of which are a change in the ability to distinguish targets from nontargets (i.e., sensitivity) and a shift toward a more conservative response criterion. Again, because we did not have a manipulation of signal intensity, we could not determine how often people experienced attentional lapses, which McCarley and Yamani (2021) operationalized as errors that occur even on high signal-intensity trials.

### Limitations

The present experiments suffer from a few limitations worth noting. Because of a move between universities, Experiment 3 was delivered in a different setting than Experiments 1 and 2, and to different samples of students. We did our best to keep the experimental conditions consistent across settings (e.g., monitor size, viewing distance monitor, room lighting, experiment software) Other than the difference in fluid intelligence across samples, other participant characteristics and experimental conditions cannot be definitively ruled out.

## Conclusions

Periodic performance feedback improved vigilance performance, particularly for individuals who had relatively lower fluid intelligence. These findings cast doubt on a resource-depletion account of the vigilance decrement and are more consistent with a resource-sharing account. Further, the present findings also reveal that the affordance of feedback is particularly effective for improving the vigilance of people with lower cognitive ability.

## Supplementary Information

Below is the link to the electronic supplementary material.

Supplementary file1 (DOCX 32 KB)

## Data Availability

All data and materials are publicly available on the Open Science Framework (https://osf.io/fzd2h/).

## Footnotes

1 Several different dependent variables can be computed to measure sensitivity. Two alternatives are *A* and *A′*. Both *A* (*r* = .91) and *A′* (*r* = .92) were highly correlated with *d′*. Therefore, we only report analyses on *d′.*

2 The end-of-task feedback was part of the design for Campbell et al. (2026a), who were interested in whether informing participants about their performance relative to their peers affected either performance on measures of cognitive ability or the latent factor structure of cognitive abilities. The performance distribution information used to provide percentile scores was taken from the mean and standard deviation in AVT performance reported by Unsworth et al. (2009).

3 We checked the robustness of these adjustments against a log-linear correction (Hautus, 1995). Our corrected hit and false-alarm rates correlated with a log-linear adjusted rates at .98 and .99, respectively. Therefore, we retained our current adjustments and report the analyses on those.

4 In our preregistration, we noted that we would exclude participants with a “sensitivity less than 0,” by which we meant an unstandardized false-alarm rate greater than an unstandardized hit rate.

5 Means and standard deviations for *d′*, hit rate, false-alarm rate, and criterion (*c*) for all experiments, broken down by condition, are listed in the Appendix.

6 To assess whether the changes in sensitivity would be modeled better by a nonlinear function, we also compared a linear fit to a quadratic fit. The linear model fit the data better than the quadratic model based on a BIC comparison (BF_10_ > 100,000).

7 This is a slightly smaller effect size than that used in our power analysis for Experiment 2 because we used the effect size from the Block × Feedback interaction on *d′* for the power analysis. With the achieved sample size after exclusions, we had about .85 power to detect this difference in Experiment 2.

8 We used a different technique (per-participant slopes) here than what is reported in Experiment 1 (Block 1 sensitivity − Block 5 sensitivity). See Footnote 4.

9 The linear model again fit the data much better than a quadratic fit (BF_10_ > 100,000).

10 Between administering Experiments 2 and 3, author Robison’s lab relocated from UTA to the University of Notre Dame. That is why the samples come from human subject pools at different universities.

11 Many of these participants were excluded because the task was crashing after 5 blocks for participants in this condition due to a programming error. The error was corrected after the first week of data collection.

12 Number series and letter sets correlated at *r* = .46; number series and Raven at *r* = .44; and letter sets and Raven at *r* = .43.

## Code availability

All analysis code is publicly available on the Open Science Framework (https://osf.io/fzd2h/).

## Declarations

### Open practices statement

The target sample size for Experiment 1 was specified for larger study from which these data are a subset (Campbell et al., 2026a). That study was preregistered on the Open Science Framework prior to data collection (https://osf.io/nc8g9). However, the analysis plan and hypotheses for the time-on-task analyses examined here were not part of that preregistration. For Experiment 2, the design, target sample size, hypotheses, and analysis procedure were preregistered prior to data collection (https://osf.io/fzd2h/). For Experiment 3, the design, hypothesis, and analysis plan were preregistered prior to data collection (https://osf.io/bwk4y). The trial-level data for all three experiments and the analysis code are available in the same repository on the Open Science Framework (https://osf.io/fzd2h).
