# Supplementary material for: Periodic performance feedback and cognitive ability interactively mitigate the vigilance decrement
Source: Atten Percept Psychophys. 2026 Aug 20;88(7):180. doi: 10.3758/s13414-026-03314-8 (PMC13493477; doi:10.3758/s13414-026-03314-8)
Supplement: Supplementary file 1 [file 13414_2026_3314_MOESM1_ESM.docx]

**Appendix**

**Table A1**

*Dependent variables by block and condition in Experiment 1*

|  |  | **Condition** | | | | |
| --- | --- | --- | --- | --- | --- | --- |
|  |  |  | |  |  | |
|  |  | ***Control*** | |  | ***Feedback*** | |
|  |  |  |  |  |  |  |
| **Measure** | **Block** | **Mean** | ***SD*** |  | **Mean** | ***SD*** |
| Sensitivity (*d’*) | 1 | 3.46 | 1.27 |  | 3.43 | 1.43 |
|  | 2 | 3.26 | 1.27 |  | 3.67 | 1.26 |
|  | 3 | 3.05 | 1.39 |  | 3.53 | 1.22 |
|  | 4 | 2.90 | 1.37 |  | 3.45 | 1.29 |
|  | 5 | 2.77 | 1.36 |  | 3.37 | 1.25 |
| Hit rate | 1 | 0.85 | 0.23 |  | 0.84 | 0.27 |
|  | 2 | 0.83 | 0.22 |  | 0.88 | 0.23 |
|  | 3 | 0.78 | 0.25 |  | 0.85 | 0.23 |
|  | 4 | 0.75 | 0.27 |  | 0.84 | 0.23 |
|  | 5 | 0.72 | 0.27 |  | 0.83 | 0.23 |
| False alarm rate | 1 | 0.03 | 0.05 |  | 0.03 | 0.08 |
|  | 2 | 0.03 | 0.06 |  | 0.02 | 0.06 |
|  | 3 | 0.04 | 0.08 |  | 0.02 | 0.05 |
|  | 4 | 0.04 | 0.08 |  | 0.03 | 0.10 |
|  | 5 | 0.04 | 0.07 |  | 0.03 | 0.07 |
| Criterion (*c*) | 1 | -0.30 | 0.45 |  | -0.31 | 0.58 |
|  | 2 | -0.36 | 0.41 |  | -0.28 | 0.52 |
|  | 3 | -0.43 | 0.48 |  | -0.34 | 0.50 |
|  | 4 | -0.48 | 0.48 |  | -0.34 | 0.51 |
|  | 5 | -0.55 | 0.50 |  | -0.40 | 0.46 |

*Note. SD* = standard deviation.

**Table A2**

*Dependent variables by block and condition in Experiment 2*

|  |  | **Condition** | | | | |
| --- | --- | --- | --- | --- | --- | --- |
|  |  |  | |  |  | |
|  |  | ***Control*** | |  | ***Feedback*** | |
|  |  |  |  |  |  |  |
| **Measure** | **Block** | **Mean** | ***SD*** |  | **Mean** | ***SD*** |
| Sensitivity (*d’*) | 1 | 3.41 | 1.15 |  | 3.34 | 1.28 |
|  | 2 | 2.99 | 1.02 |  | 3.50 | 1.13 |
|  | 3 | 2.76 | 1.25 |  | 3.31 | 1.23 |
|  | 4 | 2.47 | 1.22 |  | 3.06 | 1.04 |
|  | 5 | 2.42 | 1.22 |  | 3.17 | 1.06 |
|  | 6 | 2.21 | 1.08 |  | 3.01 | 1.24 |
|  | 7 | 2.39 | 1.20 |  | 2.92 | 0.93 |
|  | 8 | 2.40 | 1.13 |  | 3.13 | 1.05 |
|  | 9 | 2.32 | 1.24 |  | 3.07 | 1.14 |
|  | 10 | 2.24 | 1.21 |  | 2.82 | 1.21 |
| Hit rate | 1 | 0.87 | 0.16 |  | 0.88 | 0.17 |
|  | 2 | 0.78 | 0.21 |  | 0.90 | 0.13 |
|  | 3 | 0.74 | 0.24 |  | 0.88 | 0.13 |
|  | 4 | 0.69 | 0.23 |  | 0.82 | 0.15 |
|  | 5 | 0.66 | 0.26 |  | 0.83 | 0.17 |
|  | 6 | 0.65 | 0.22 |  | 0.80 | 0.18 |
|  | 7 | 0.64 | 0.25 |  | 0.78 | 0.18 |
|  | 8 | 0.67 | 0.24 |  | 0.83 | 0.16 |
|  | 9 | 0.63 | 0.24 |  | 0.80 | 0.19 |
|  | 10 | 0.62 | 0.24 |  | 0.80 | 0.17 |
| False alarm rate | 1 | 0.03 | 0.04 |  | 0.03 | 0.03 |
|  | 2 | 0.03 | 0.03 |  | 0.03 | 0.04 |
|  | 3 | 0.04 | 0.04 |  | 0.04 | 0.06 |
|  | 4 | 0.05 | 0.06 |  | 0.03 | 0.04 |
|  | 5 | 0.04 | 0.04 |  | 0.04 | 0.05 |
|  | 6 | 0.06 | 0.06 |  | 0.04 | 0.05 |
|  | 7 | 0.04 | 0.04 |  | 0.03 | 0.03 |
|  | 8 | 0.04 | 0.04 |  | 0.04 | 0.05 |
|  | 9 | 0.05 | 0.06 |  | 0.03 | 0.03 |
|  | 10 | 0.05 | 0.06 |  | 0.05 | 0.07 |

*Note. SD* = standard deviation.

**Table A2 (*continued*)**

*Dependent variables by block and condition in Experiment 2*

|  |  | **Condition** | | | | |
| --- | --- | --- | --- | --- | --- | --- |
|  |  |  | |  |  | |
|  |  | ***Control*** | |  | ***Feedback*** | |
|  |  |  |  |  |  |  |
| **Measure** | **Block** | **Mean** | ***SD*** |  | **Mean** | ***SD*** |
| Criterion (*c*) | 1 | -0.23 | 0.38 |  | -0.18 | 0.37 |
|  | 2 | -0.43 | 0.49 |  | -0.23 | 0.37 |
|  | 3 | -0.47 | 0.45 |  | -0.29 | 0.36 |
|  | 4 | -0.55 | 0.43 |  | -0.39 | 0.36 |
|  | 5 | -0.59 | 0.48 |  | -0.33 | 0.45 |
|  | 6 | -0.62 | 0.37 |  | -0.38 | 0.36 |
|  | 7 | -0.68 | 0.40 |  | -0.50 | 0.37 |
|  | 8 | -0.60 | 0.44 |  | -0.41 | 0.42 |
|  | 9 | -0.64 | 0.44 |  | -0.42 | 0.37 |
|  | 10 | -0.68 | 0.44 |  | -0.38 | 0.38 |

*Note. SD* = standard deviation.

**Table A3**

*Dependent variables by block and condition in Experiment 3*

|  |  | **Condition** | | | | | |
| --- | --- | --- | --- | --- | --- | --- | --- |
|  |  |  | |  | |  | |
|  |  | ***Whole Task*** | | ***First Half*** | | ***No Feedback*** | |
|  |  |  |  |  |  |  |  |
| **Measure** | **Block** | **Mean** | ***SD*** | **Mean** | ***SD*** | **Mean** | ***SD*** |
| Sensitivity (*d'*) | 1 | 4.26 | 0.52 | 4.12 | 0.55 | 4.18 | 0.61 |
|  | 2 | 4.27 | 0.58 | 4.39 | 0.49 | 4.29 | 0.59 |
|  | 3 | 4.22 | 0.63 | 4.29 | 0.53 | 4.05 | 0.85 |
|  | 4 | 4.04 | 0.80 | 4.09 | 0.73 | 4.09 | 0.69 |
|  | 5 | 3.94 | 0.81 | 3.96 | 0.84 | 4.01 | 0.80 |
|  | 6 | 4.09 | 0.64 | 4.06 | 0.77 | 4.13 | 0.67 |
|  | 7 | 3.84 | 0.87 | 3.91 | 0.79 | 3.76 | 1.08 |
|  | 8 | 3.86 | 0.93 | 3.76 | 0.82 | 3.73 | 0.93 |
|  | 9 | 3.96 | 0.75 | 3.79 | 0.90 | 3.65 | 0.99 |
|  | 10 | 3.71 | 0.95 | 3.61 | 1.05 | 3.71 | 1.01 |
| Hit Rate | 1 | 0.97 | 0.03 | 0.96 | 0.04 | 0.96 | 0.06 |
|  | 2 | 0.96 | 0.05 | 0.98 | 0.03 | 0.96 | 0.07 |
|  | 3 | 0.97 | 0.04 | 0.96 | 0.05 | 0.94 | 0.09 |
|  | 4 | 0.93 | 0.12 | 0.95 | 0.08 | 0.95 | 0.07 |
|  | 5 | 0.93 | 0.09 | 0.92 | 0.11 | 0.93 | 0.09 |
|  | 6 | 0.95 | 0.06 | 0.94 | 0.08 | 0.96 | 0.06 |
|  | 7 | 0.90 | 0.12 | 0.92 | 0.10 | 0.90 | 0.14 |
|  | 8 | 0.91 | 0.13 | 0.91 | 0.10 | 0.89 | 0.15 |
|  | 9 | 0.91 | 0.11 | 0.90 | 0.13 | 0.89 | 0.14 |
|  | 10 | 0.88 | 0.15 | 0.87 | 0.16 | 0.89 | 0.14 |
| False Alarm Rate | 1 | 0.02 | 0.01 | 0.02 | 0.01 | 0.02 | 0.01 |
|  | 2 | 0.01 | 0.00 | 0.01 | 0.00 | 0.01 | 0.01 |
|  | 3 | 0.01 | 0.00 | 0.01 | 0.00 | 0.01 | 0.01 |
|  | 4 | 0.01 | 0.01 | 0.01 | 0.01 | 0.01 | 0.01 |
|  | 5 | 0.01 | 0.01 | 0.01 | 0.01 | 0.01 | 0.01 |
|  | 6 | 0.01 | 0.00 | 0.01 | 0.01 | 0.01 | 0.01 |
|  | 7 | 0.01 | 0.01 | 0.01 | 0.01 | 0.02 | 0.04 |
|  | 8 | 0.01 | 0.01 | 0.02 | 0.01 | 0.02 | 0.01 |
|  | 9 | 0.01 | 0.01 | 0.02 | 0.01 | 0.02 | 0.02 |
|  | 10 | 0.02 | 0.01 | 0.02 | 0.01 | 0.02 | 0.01 |

*Note. SD* = standard deviation.

**Table A3 (*continued*)**

*Dependent variables by block and condition in Experiment 3*

|  |  | **Condition** | | | | | |
| --- | --- | --- | --- | --- | --- | --- | --- |
|  |  |  |  |  |  |  |  |
|  |  | ***Whole Task*** | | ***First Half*** | | ***No Feedback*** | |
|  |  |  |  |  |  |  |  |
| **Measure** | **Block** | **Mean** | ***SD*** | **Mean** | ***SD*** | **Mean** | ***SD*** |
| Criterion (*c*) | 1 | -0.06 | 0.20 | -0.09 | 0.27 | -0.11 | 0.24 |
|  | 2 | -0.14 | 0.27 | -0.08 | 0.18 | -0.13 | 0.28 |
|  | 3 | -0.15 | 0.27 | -0.13 | 0.23 | -0.17 | 0.26 |
|  | 4 | -0.23 | 0.37 | -0.19 | 0.30 | -0.18 | 0.29 |
|  | 5 | -0.26 | 0.33 | -0.25 | 0.35 | -0.20 | 0.31 |
|  | 6 | -0.21 | 0.30 | -0.21 | 0.31 | -0.16 | 0.28 |
|  | 7 | -0.30 | 0.36 | -0.27 | 0.35 | -0.27 | 0.35 |
|  | 8 | -0.26 | 0.33 | -0.29 | 0.34 | -0.30 | 0.36 |
|  | 9 | -0.28 | 0.36 | -0.30 | 0.40 | -0.28 | 0.37 |
|  | 10 | -0.33 | 0.39 | -0.35 | 0.37 | -0.31 | 0.37 |

*Note. SD* = standard deviation.
